# Facile synthesis of 7-alkyl-1,2,3,4-tetrahydro-1,8-naphthyridines as arginine mimetics using a Horner–Wadsworth–Emmons-based approach

**DOI:** 10.3762/bjoc.16.134

**Published:** 2020-07-08

**Authors:** Rhys A Lippa, John A Murphy, Tim N Barrett

**Affiliations:** 1 Department of Pure & Applied Chemistry, University of Strathclyde, 295 Cathedral Street, Glasgow G1 1XL, Scotland, U.K.; 2 GlaxoSmithKline Medicines Research Centre, Gunnels Wood Road, Stevenage SG1 2NY, U.K.

**Keywords:** arginine, Horner–Wadsworth–Emmons, integrin, phosphoramidate, tetrahydronaphthyridine

## Abstract

Integrin inhibitors based on the tripeptide sequence Arg–Gly–Asp (RGD) are potential therapeutics for the treatment of idiopathic pulmonary fibrosis (IPF). Herein, we describe an expeditious three-step synthetic sequence of Horner–Wadsworth–Emmons olefination, diimide reduction, and global deprotection to synthesise cores for these compounds in high yields (63–83% over 3 steps) with no need for chromatography. Key to this transformation is the phosphoramidate protecting group, which is stable to metalation steps.

## Introduction

**Figure 1:**
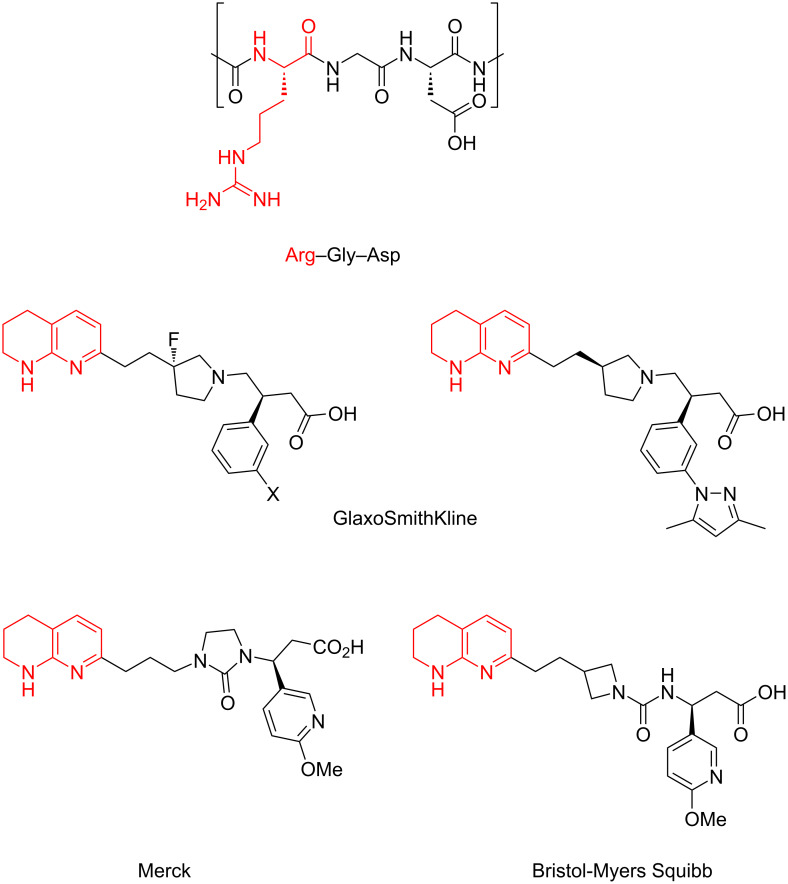
The Arg–Gly–Asp tripeptide sequence and examples of tetrahydro-1,8-naphthyridine-containing integrin inhibitors.

Tetrahydronaphthyridines are prominent in peptidomimetic pharmaceuticals as arginine mimetics and they are widely used in Arg–Gly–Asp (RGD) peptide mimetics such as αv integrin inhibitors [1]. Tetrahydronaphthyridines represent less basic but more permeable alternatives to arginine (p*K*_a_ ≈ 7 versus 13.8) [1] replicating the side-on salt-bridge binding interaction made between the guanidinium functionality of arginine and an aspartic acid residue in the protein. Consequently, this moiety has been used in various integrin inhibitors (Figure 1) [2–8].

**Scheme 1:**
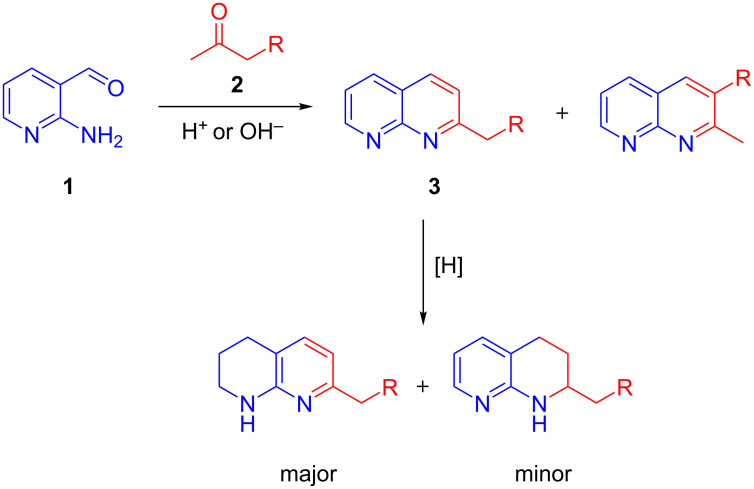
Commonly used synthetic routes to tetrahydro-1,8-naphthyridine moieties by hydrogenation of saturated naphthyridines **3**.

Current routes to install tetrahydronaphthyridines predominantly revolve around late-stage hydrogenation of fully aromatic 1,8-naphthyridine derivatives **3**, usually prepared via an acid or base-catalysed Friedländer reaction between 2-aminonicotinaldehyde (**1**) and the corresponding ketone **2** (Scheme 1). Both reactions employ harsh conditions with limited functional group tolerance and limited regiochemical control, which presents considerable purification issues during large-scale synthesis [8–10]. More recently, catalytic methodologies for the asymmetric hydrogenation of 1,8-naphythyridines have been reported [11–12].

**Scheme 2:**
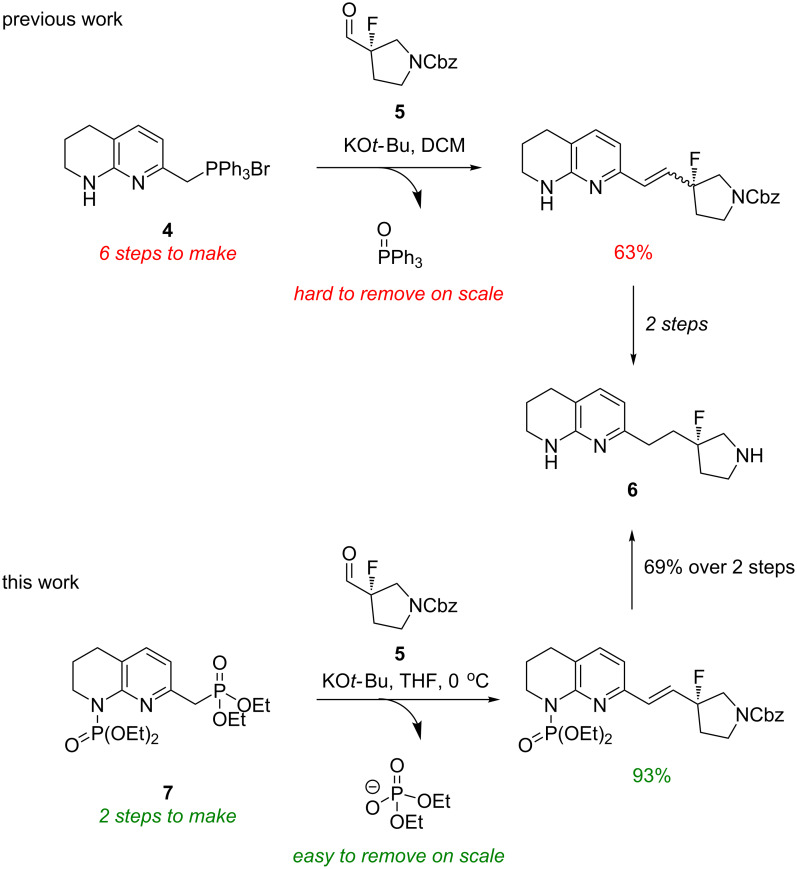
Previous synthetic route to fluoropyrrolidine **6** utilising a Wittig reaction and the novel, higher yielding route using a Horner–Wadsworth–Emmons reaction.

Recently, GlaxoSmithKline disclosed a route to a fluoropyrrolidine **6** using a Wittig reaction between phosphonium salt **4** and aldehyde **5** [2]. The synthesis of phosphonium salt **4** (itself requiring 6 steps including partial saturation of a 1,8-naphthyridine moiety) and the formation of the triphenylphosphine oxide byproduct in the Wittig step presented complications on both gram and kilogram scales. Herein, we report a novel synthetic sequence to tetrahydro-1,8-naphthyridines using a Horner–Wadsworth–Emmons reaction using diphosphorylated compound **7**, proceeding in high yields and high purities without the need for chromatographic purification (Scheme 2).

## Results and Discussion

**Scheme 3:**
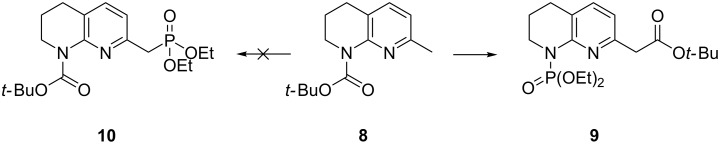
Synthesis of phosphoramidate **9** from tetrahydro-1,8-naphthyridine **8**. Conditions: *s*-BuLi (3 equiv), diethyl chlorophosphate (1.1 equiv), THF, −42 °C, 30 min, 14% yield.

Based on applications of phosphonates derived from 2-methylpyridine and 2-methlylquinoline derivatives in Horner–Wadsworth–Emmons olefinations [13–15], it was proposed that similar a methodology could be applied to 7-methyl-1,2,3,4-tetrahydro-1,8-naphthyridine analogues. The investigation initially began using commercially available *N*-Boc-protected tetrahydro-1,8-naphthyridine **8** [16]. Upon deprotonation and quenching with diethyl chlorophosphate, migration of the Boc group from the nitrogen atom to the exocyclic methyl group was observed, affording phosphoramidate **9** in low yield, indicating good leaving group ability of the stabilised tetrahydronaphthyridine anion. No formation of phosphonate **10** was detected (Scheme 3).

**Scheme 4:**
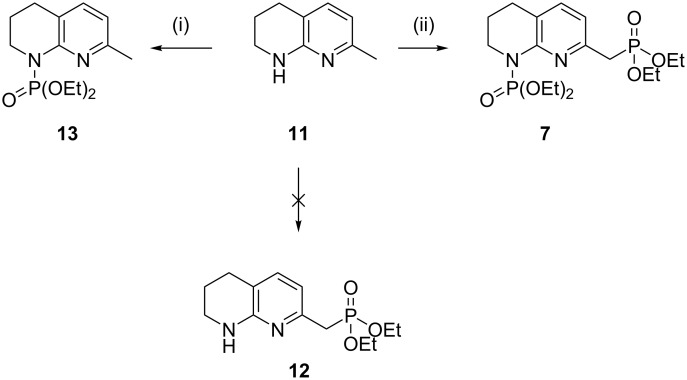
Mono- and diphosphorylation of tetrahydro-1,8-naphthyridine **11**. Conditions: (i) *s*-BuLi (2 equiv), diethyl chlorophosphate (1 equiv), THF, −78 °C, 4 h, 44% yield; (ii) *s*-BuLi (2 equiv), diethyl chlorophosphate (2 equiv), THF, −78 °C, 4 h, 33% yield.

It was proposed that a deprotonation of 7-methyl-1,2,3,4-tetrahydro-1,8-naphthyridine (**11**) with two equivalents of *sec*-BuLi would afford phosphonate **12** upon quenching with diethyl chlorophosphate via formation of the dianion. This could then be used in a subsequent Horner–Wadsworth–Emmons reaction to construct the carbon skeleton of amine **6**. Upon the addition of a single equivalent of diethyl chlorophosphate, phosphoramidate **13** was obtained exclusively at both −42 and −78 °C. The addition of two equivalents of the chlorophosphate yielded diphosphorylated compound **7**, albeit in poor yield (Scheme 4).

**Scheme 5:**
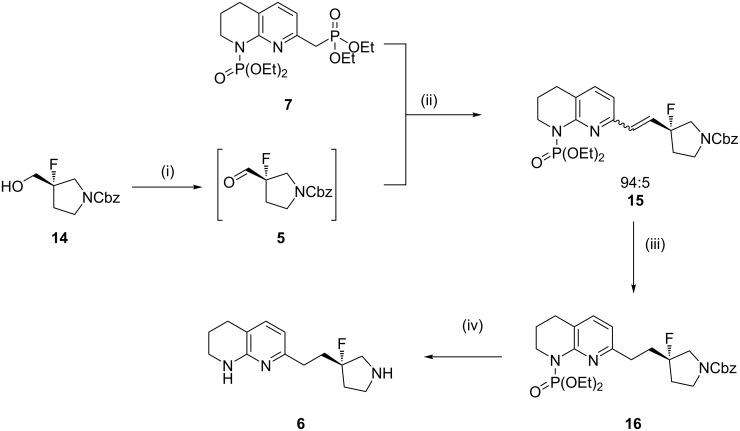
Synthesis of amine **6** from phosphonate **7** and aldehyde **5**. Conditions: (i) T3P^®^ (50% w/w in DCM, 3 equiv), DMSO (3 equiv), DIPEA (2.5 equiv), DCM, 0 °C, 1 h; (ii) KO*t-*Bu (6 equiv), THF, 0 °C, 70 min, 93% yield over two steps (relative to phosphonate **7**); (iii) PhSO_2_NHNH_2_ (3 equiv), K_2_CO_3_ (4 equiv), DMF, 100 °C, 30 min, 80% yield; (iv) 7.4 M HCl, 100 °C, 1.5 h, 86% yield.

The deprotonation of phosphonate **7** and subsequent reaction with aldehyde **5**, formed in situ by oxidation of alcohol **14** using T3P^®^ [17], yielded olefin **15** in 93% yield as a 94:5 mixture of stereoisomers – presumably *E*/*Z*, although this is not conclusive from the ^1^H NMR spectrum. Based on LC–MS and ^1^H NMR spectroscopy observations, it is believed that aldehyde **5** exists predominantly as the corresponding hydrate and an excess of base (6 equiv) was required to overcome stalling of the olefination process. Reduction to compound **16** using benzenesulfonyl hydrazide (generating diimide in situ) proceeded in 80% yield and was followed by single-pot carbamate and phosphonate deprotection to afford arginine mimetic **6** in 86% yield. This represents a 64% overall yield which was increased to 68% when no column chromatography was undertaken between transformations, with no loss of purity (Scheme 5).

Olefin reduction and Cbz deprotection could not be performed simultaneously by palladium-catalysed hydrogenation as this resulted in defluorination, presumably via a Tsuji–Trost-like elimination of the allylic fluoride [18–19]. This sequence represents a marked improvement from the Wittig-including route, lowering the number of synthetic steps and increasing the overall yield [2]. Furthermore, no problematic byproducts are formed, and good purity is obtained without the use of any chromatography, which is ideal for large-scale processes.

### Optimisation of the synthesis of phosphoramidate **13** and phosphonate **7**

Having been shown to be a feasible intermediate, attention turned to improving the synthesis of bisphosphonate **7** via a two-step process, exploiting the base stability of the phosphoramidate protecting group. A variety of bases were trialed at 0 °C for the initial *N*-phosphorylation, with 10 minutes allowed for complete deprotonation to occur (Table 1).

**Table 1:** Bases surveyed for the formation of phosphoramidate **13**.^a^

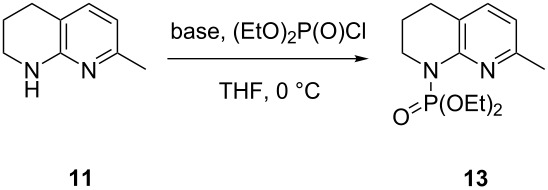		

Entry	Base	Amount of phosphoramidate **13**/LC–MS a/a%

1	KO*t-*Bu (1 M in THF)	4
2	LiHMDS (1 M in THF)	58
3	LDA (2 M in hexanes/benzene)	58
4	*s-*BuLi (1.4 M in cyclohexane)	65
5	iPrMgCl (2 M in THF)	88

^a^Reactions performed on a 0.7 mmol scale of compound **11**.

Only minimal phosphorylation was observed when using potassium *tert*-butoxide (Table 1, entry 1). This may be due the disparity in the p*K*_a_ between the base and tetrahydro-1,8-naphthyridine (based on 2-aminopyridine, the p*K*a of the saturated ring nitrogen is expected to be ≈28) [20]. Similarly, nitrogen-centred bases (Table 1, entries 2 and 3) gave moderate conversions to phosphoramidate **13** due to a close match of the p*K*_a_ of tetrahydronaphthyridine **11** and the p*K*_aH_ of the base; *s*-BuLi also gave reasonable conversion to the product (Table 1, entry 4) albeit contaminated with some impurities. This could likely be attributed to the thermal instability of the base and/or lithiated tetrahydronaphthyridine in THF at this temperature. The use of iPrMgCl, a strong but room temperature-stable base, gave the highest conversion to phosphoramidate **13**. Further investigation into the use of iPrMgCl revealed that a quantitative conversion was achieved at ambient temperature with a metalation time of <1 minute. Pleasingly, premixing of tetrahydronaphthyridine **11** with diethyl chlorophosphate, followed by dropwise addition of iPrMgCl gave a clean and total conversion to phosphoramidate **13** as seen by LC–MS. When performed on a multigram scale, a 94% yield of compound **13** was obtained. The reaction also proceeded well (91% isolated yield) in 2-MeTHF, which offers a preferred alternative if the reaction is performed on a larger scale due to better partitioning with water, stability, and sustainability of production [21]. Of the bases trialled, only *s*-BuLi was efficient in promoting *C*-phosphorylation. Optimisation of the use of this base was then investigated further (Table 2).

**Table 2:** Optimisation of the formation of phosphonate **7** using *s*-BuLi as a base.^a^

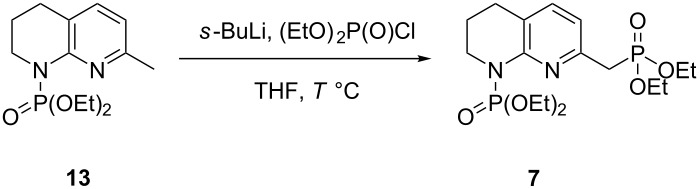				

Entry	*T*/°C	*s*-BuLi/equiv	Lithiation time/min	Amount of phosphonate **7**/LC–MS a/a%

1	−78	1.5	90	53
2	−42	2.0	20	62
3	−42	2.5	20	81
4	−42	3.0	20	86
5	−42	3.5	20	67
6	−42	4.0	20	48
7	−42	3.0 (+ 3 equiv TMEDA)	20	59
8	0	2.0	10	0

^a^Reactions were performed on a 4.6 mmol scale (entry 1), 0.5 mmol scale (entries 2–6), 0.3 mmol (entry 7), or 0.4 mmol scale (entry 8).

**Scheme 6:**
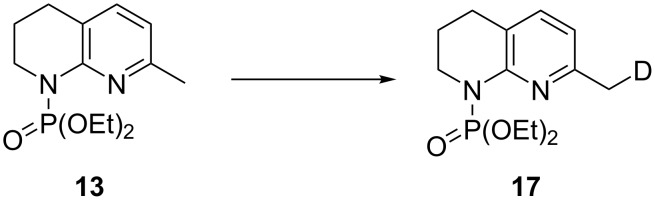
Monodeuteration of **13** as observed by ^1^H and ^13^C NMR. Conditions: *s*-BuLi (3 equiv), THF, −42 °C, 20 min then CD_3_OD (14 equiv), 5 min.

An optimal deprotonation and phosphorylation was found to occur when an excess (3 equiv) of base was used at −42 °C (Table 2, entry 4), with a lithiation time of 20 min. The addition of TMEDA was detrimental to this conversion with a large proportion of starting material **13** remaining (Table 2, entry 7). No product was formed at higher temperatures (Table 2, entry 8), likely due to the instability of the *C*-lithiated species at elevated temperatures as degradation was observed. It is believed that the excess base loading is required to account for the deprotonation of the more acidic phosphonate product **7** versus the starting material **13**, and potential lithium sequestration by chelation between an oxygen atom of the phosphonate and the nitrogen atom of the unsaturated ring. Monitoring of the deprotonation followed by quenching with CD_3_OD by ^1^H and ^13^C NMR spectroscopy indicated the formation of the monolabelled compound **17** as the major product, demonstrating that lithiation only occurs at a single position of compound **13** (Scheme 6).

**Scheme 7:**
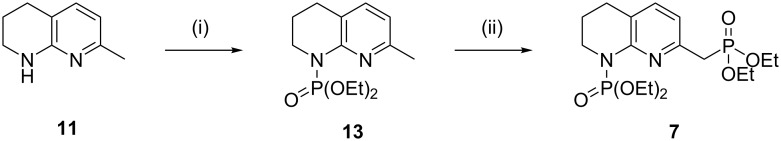
Sequential diphosphorylation of tetrahydronaphthyridine **11**. Conditions: (i) iPrMgCl (1.5 equiv), THF, then diethyl chlorophosphate (1.2 equiv), 5 min, 94% yield; (ii) *s*-BuLi (3 equiv), THF, −42 °C, 20 min, then (EtO)_2_P(O)Cl (1.1 equiv), 20 min, 68% yield.

When performed on a multigram scale, phosphonate **7** was isolated in 68% yield after purification by chromatography on silica. This, combined with the formation of phosphoramidate **13** in 94% yield, represented a marked improvement to the initial one-pot diphosphorylation (Scheme 7). Contrary to reports using 2-methylquinolines, tandem phosphorylation and olefination was not possible [15]. When a sequential diphosphorylation was attempted in a single pot, diphosphorylated compound **7** was not observed, with phosphoramidate **13** accounting for the majority of product formed [22].

### Reaction scope

The sequence of olefination, reduction, and deprotection was tested on other arginine mimetics of varying amine structure, constituting potential Arg–Gly components of Arg–Gly–Asp integrin inhibitors (Table 3). Where the aldehyde was not commercially available, *N*-Boc-protected alcohols were oxidised using IBX in refluxing ethyl acetate and used crude. Olefination required only a modest excess of base (1.5–2.4 equiv) and proceeded in <1 h. Sodium hydride was investigated as a heterogeneous alternative to potassium *tert*-butoxide, although a lower isolated yield of the final amine was obtained. All amines were formed in high NMR and LC–MS purity without the need for purification by column chromatography.

**Table 3:** Cores synthesised by the sequence of olefination, reduction, and deprotection, and the corresponding starting alcohols and aldehydes.^a^

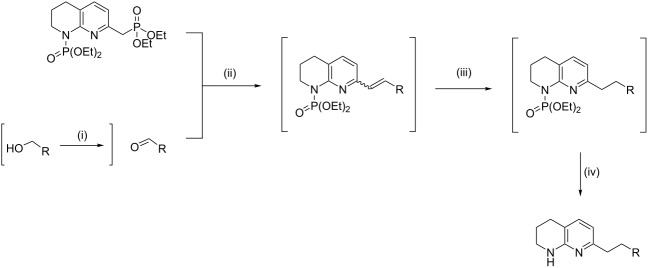				

Entry	Alcohol	Aldehyde	Product	Yield

1	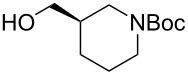 **18**	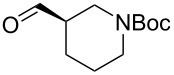 **19**	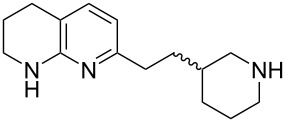 **20**	82%^b^
2	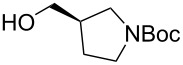 **21**	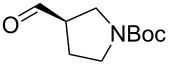 **22**	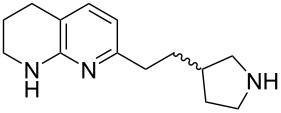 **23**	83%^b^
3	–	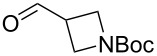 **24**	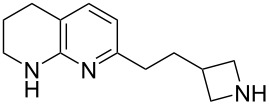 **25**	79%^c^
4	–	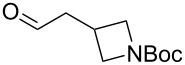 **26**	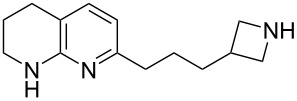 **27**	63%^d^

^a^Conditions: ^b^(i) IBX (3 equiv), EtOAc, reflux, 2.25–3 h; (ii) KO*t-*Bu (1.5–2.3 equiv), THF, 0 °C, 7–40 min; K_2_CO_3_ (4 equiv), PhSO_2_NHNH_2_ (3 equiv), DMF, 100 °C, 70–80 min; (iv) 7.4 M HCl, 100 °C, 2 h; ^c^(ii) KO*t-*Bu (1.8 equiv), THF, 0 °C, 5 min; (iii) K_2_CO_3_ (4 equiv), PhSO_2_NHNH_2_ (3 equiv), DMF, 100 °C, 75 min; (iv) 7.4 M HCl, 100 °C 3.5 h; ^d^(ii) NaH (2.4 equiv), THF, 0 °C, 70 min; (iii) K_2_CO_3_ (4 equiv), PhSO_2_NHNH_2_ (3 equiv), DMF, 100 °C, 2 h; (iv) 7.4 M HCl, 100 °C, 2 h.

Pleasingly, the sequence proceeded in high yields (63–83%) for all substrates with no chromatography required. Despite relatively high yields, significant racemisation was seen in the synthesis of piperidine **20** and pyrrolidine cores **23**. Pyrrolidine **23** was obtained with an ee of only 30%, representing a significant loss of enantiopurity. It is believed that racemisation occurred during the olefination step, caused by a base mediated keto–enol tautomerisation. It was proposed that the use of lithium hydroxide would promote hydrate formation, mitigating racemisation of the aldehyde. However, this was unsuccessful as complete racemisation was observed, with olefination requiring five days to complete. No olefination was seen using DIPEA under refluxing conditions. As such, this sequence, as currently performed, is suitable for substrates lacking an acidic α-proton (fluoropyrrolidine core **6**) or achiral aldehydes (azetidine cores **25** and **27**).

**Scheme 8:**
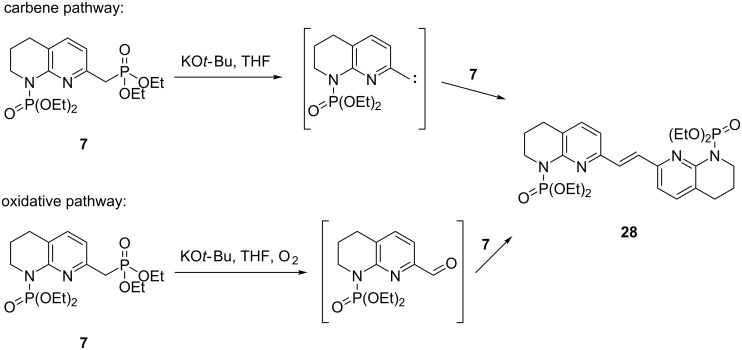
Possible mechanistic pathways for the formation of dimer **28**. Conditions: KO*t-*Bu, THF, 1 h, 68% yield.

Furthermore, key to success of the Horner–Wadsworth–Emmons olefination was premixing of the aldehyde and phosphonate **7** prior to the addition of KO*t-*Bu. Upon deprotonation, phosphonate **7** (in the absence of aldehyde) underwent dimerisation to olefin **28**. While the exact mechanism is not known, it may involve a reactive carbene intermediate formed by α-elimination of the phosphonate as described in previous reports [23]. Alternatively, a base-promoted autoxidation of phosphonate **7** in the presence of trace amounts of oxygen, followed by olefination may be taking place (Scheme 8) [24–26]. The autoxidation of phosphonates under THF/potassium *tert*-butoxide has been reported previously [27].

**Scheme 9:**
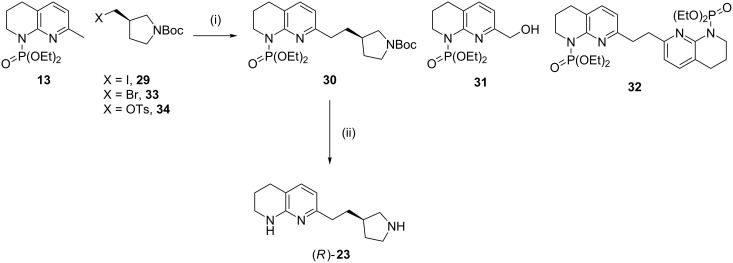
Alkylation of phosphoramidate **13** by iodide **29** to afford compound **30** and byproducts alcohol **31** and dimer **32**. Use of bromide **33** or tosylate **34** afforded only compounds **31** and **32**. Conditions: (i) *s*-BuLi (1.3 equiv), iodide **29**, THF, −78 °C, 30 min; (ii) 7.4 M HCl, 100 °C, 3.5 h, 92% yield.

In order to circumvent racemisation of aldehyde **22** during the Horner–Wadsworth–Emmons olefination, alkylation of phosphoramidate **13** was explored using commercially available iodide **29**. The formation of compound **30** proceeded in 21% yield, with alcohol **31** and dimer **32** also formed in 20% and 5% yield, respectively (Scheme 9). Indeed, when iodide **29** was replaced with bromide **33** and tosylate **34** no formation of compound **30** was observed, with alcohol **31** and dimer **32** accounting for the major products. Acidic deprotection of phosphoramidate **30** afforded amine (*R*)*-***23** in 92% yield in >99% ee, offering an alternative route to the Horner–Wadsworth–Emmons-based approach.

The mechanism of the formation of alcohol **31** and dimer **32** was not fully explored although it is likely to occur via a radical pathway. When iodide **29** was replaced by a superior oxidant in 1,2-dibromoethane, formation of dimer **32** increased (17% isolated yield). This supports the previously reported proposals that dimerisation occurs via single-electron oxidation of the 2-picolyl anion by the halide/pseudohalide oxidant followed by recombination of the resulting picolyl radical [28–30]. Whilst trace oxygen may be involved, as indicated by the presence of alcohol **31**, no dimerisation or alcohol formation was seen in the absence of a halide/pseudohalide-based oxidant, during *C*-phosphorylation or deuteration.

## Conclusion

In conclusion, a novel method for the assembly of 7-alkyl-1,2,3,4-tetrahydro-1,8-naphthyridine-based arginine mimetics was developed. The synthesis of phosphonate **7** was optimised, with a sequential diphosphorylation process using commercially available starting materials affording the desired compound in 64% overall yield. A Horner–Wadsworth–Emmons/reduction/deprotection procedure was used to synthesise amines in good yield requiring no chromatography, although racemisation was observed where chiral aldehydes possessed an α-proton. This methodology utilised the underused base-stable phosphoramidate protecting group, which was superior to the more commonly applied Boc protecting group which was unstable to the lithiation. This synthetic route replaces traditional Wittig and tandem alkylation/reduction methodologies, which suffer from complications arising from troublesome byproducts and reaction selectivity. The new procedure proceeds in a higher yield than previously obtained and may potentially offer benefits in large-scale manufacture of integrin inhibitors and other arginine peptidomimetics.

## Supporting Information

File 1 Detailed experimental procedures, and product characterisation data, along with ^1^H and ^13^C NMR spectra.
