# Supplementary material for: Facile synthesis of 7-alkyl-1,2,3,4-tetrahydro-1,8-naphthyridines as arginine mimetics using a Horner–Wadsworth–Emmons-based approach
Source: Beilstein J Org Chem. 2020 Jul 8;16:1617–26. doi: 10.3762/bjoc.16.134 (PMC7356399; doi:10.3762/bjoc.16.134)
Supplement: File 1 — Detailed experimental procedures, and product characterisation data, along with 1H and 13C NMR spectra. [file Beilstein_J_Org_Chem-16-1617-s001.pdf]

BEILSTEIN JOURNAL OF ORGANIC CHEMISTRY

Supporting Information

for

Facile synthesis of 7-alkyl-1,2,3,4-tetrahydro-1,8-naphthyridines as arginine mimetics using a Horner–Wadsworth–Emmons-based approach

Rhys A. Lippa, John A. Murphy and Tim N. Barrett

Beilstein J. Org. Chem. **2020,** *16,* 1617–1626. doi:10.3762/bjoc.16.134

Detailed experimental procedures, and product characterisation data, along with ^{1}H and ^{13}C NMR spectra

License and Terms: This is a supporting information file under the terms of the Creative Commons Attribution License (http://creativecommons.org/licenses/by/4.0). Please note that the reuse, redistribution and reproduction in particular requires that the authors and source are credited.
The license is subject to the *Beilstein Journal of Organic Chemistry* terms and conditions: (https://www.beilstein-journals.org/bjoc)

Table of contents

Experimental....................S2

General points....................S2

Compound synthesis and characterisation S4

References....................S21

^{1}H and ^{13}C NMR spectra of compounds S22

Chiral HPLC analysis of compound **23** and (*R*)**-23** S36

Experimental

General points

Starting materials and all solvents were of analytical grade purchased from commercial sources. Solvents were purchased in anhydrous form unless stated otherwise. All reactions were stirred magnetically. Air and moisture-sensitive reactions were performed using standard Schlenk manifold techniques. Reagents were used as supplied unless otherwise stated. Column chromatography was performed on disposable normal-phase and C18 reversed-phase RediSep Rf columns (12–220 g). Melting points were recorded using a Büchi Melting Point apparatus M-565. Infrared spectra were recorded using a Perkin Elmer FTIR spectrometer as neat or film in the range 4000–600 cm^{-1}. Nuclear magnetic resonance spectra were recorded in the solvent stated on Bruker NMR spectrometers (AVIII 400MHz, AVIIIHD 600 MHz and AVIII 700 MHz). Spectra were referenced to the residual solvent peak. Chemical shifts (δ) are quoted in parts per million (ppm) to the nearest 0.01 ppm for ^{1}H NMR spectroscopy and 0.1 ppm for ^{13}C, and ^{31}P NMR spectroscopy. Coupling constants (*J*) are quoted in hertz (Hz) to the nearest 0.1 Hz. 2D NMR spectra were obtained to confirm structures where necessary. Multiplicity is quoted as follows: s (singlet), d (doublet), t (triplet), q (quartet), quin. (quintet), m (multiplet). Liquid chromatography mass spectra (LC–MS) were recorded using the following methods:

LC conditions:

The UPLC analysis was conducted on an Acquity UPLC CSH C18 column (50 mm x 2.1 mm i.d., 1.7 μm packing diameter) at 40 °C.

<u>HpH:</u>

2 min HpH:

The solvents employed were: A = 10 mM ammonium bicarbonate in water adjusted to pH 10 with ammonia solution, B = acetonitrile.

The gradient employed was:

Time (min)	Flow rate (mL/min)	% A	% B
0.00	1	97	3
0.05	1	97	3
1.50	1	5	95
1.90	1	5	95
2.00	1	97	3

For: 2 min

The solvents employed were: A = 0.1% v/v solution of formic acid in water adjusted, B = 0.1% v/v solution of formic acid in acetonitrile.

The gradient employed was:

Time (min)	Flow rate (mL/min)	% A	% B
0.00	1	97	3
0.05	1	97	3
1.50	1	5	95
1.90	1	5	95
2.00	1	97	3

LC–MS purities are quoted as peak area % (% a/a) seen in the chromatogram. High resolution mass spectra (HRMS) were recorded using an Acquity UPLC CSH C18 column (LC) and Waters XEVO G2-XS QTof (ES+) (MS)

Compound synthesis and characterisation

***tert*-Butyl 2-(8-(diethoxyphosphoryl)-5,6,7,8-tetrahydro-1,8-naphthyridin-2-yl)acetate (9)**

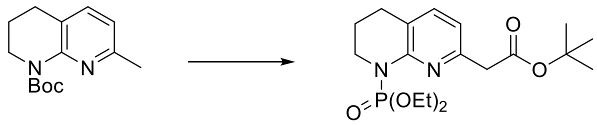

8 **9**

To a stirred solution of *tert*-butyl 7-methyl-3,4-dihydro-1,8-naphthyridine-1(2*H*)-carboxylate (**8**, 267 mg, 1.0 mmol) in THF (4 mL) under nitrogen at −42 °C, was added *s*-BuLi (1.15 M in cyclohexane, 2.6 mL, 2.99 mmol). The reaction was stirred for 20 min and then diethyl chlorophosphate (0.16 mL, 1.107 mmol) was added, and the reaction was stirred for 10 min. After this time, the reaction was quenched with sat. NH_4Cl (10 mL). The phases were separated and the aqueous phase was extracted with ethyl acetate (2 × 10 mL). The combined organics were passed through a hydrophobic frit and concentrated in vacuo. The residue was dissolved in a minimum volume of DMSO and purified by HPLC using Mass Directed AutoPrep (Xselect C18 column eluting with 30–70% acetonitrile in water with 10 mM ammonium bicarbonate modifier) to afford the title compound (55 mg, 0.143 mmol, 14% yield) as colourless gum.

^{1}H NMR (400 MHz, $CDCl_3$) δ = 7.28–7.25 (m, 1H), 6.78 (d, *J* = 7.3 Hz, 1H), 4.27–4.06 (m, 4H), 3.85–3.79 (m, 2H), 3.58 (s, 2H), 2.74 (t, *J* = 6.4 Hz, 2H), 1.96–1.88 (m, 2H), 1.44 (s, 9H), 1.28 (td, *J* = 7.1, 1.0 Hz, 6H); ^{13}C NMR (176 MHz, $CDCl_3$) δ = 170.2, 152.0 (d, *J* = 1.9 Hz), 150.6, 137.7 (d, *J* = 1.3 Hz), 117.9 (d, *J* = 7.6 Hz), 116.6, 80.8, 63.3 (d, *J* = 5.7 Hz), 46.0 (d, *J* = 2.5 Hz), 44.5, 28.0, 26.8, 22.3 (d, *J* = 4.5 Hz), 16.1 (d, *J* = 7.0 Hz); IR (neat): 2937, 2867, 1680, 1629 cm^{-1}; LC-MS (For): 1.07 min (385)

([M+H]$^+$, 96% purity a/a); HRMS: calculated for $C_{18}H_{30}N_2O_5P^+$ ([M+H]$^+$) 385.1887 found 385.1888.

Diethyl (7-methyl-3,4-dihydro-1,8-naphthyridin-1(2*H*)-yl)phosphonate (13)

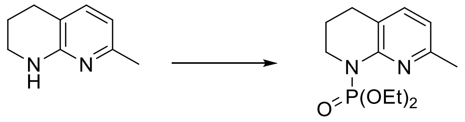

11 **13**

To a stirred solution of 7-methyl-1,2,3,4-tetrahydro-1,8-naphthyridine (**11**, 1.46 g, 9.85 mmol) in THF (35 mL) at rt was added diethyl chlorophosphate (1.70 mL, 11.76 mmol). Then, isopropylmagnesium chloride (1.42 M in THF, 10.4 mL, 14.77 mmol) was added dropwise over 5 min. After 5 min, the reaction was quenched slowly with sat. NH_4Cl (45 mL). The phases were separated and the aqueous phase was extracted with ethyl acetate (2 × 40 mL). The combined organics were washed with brine (100 mL), passed through a hydrophobic frit and concentrated in vacuo. The residue was purified using automated flash chromatography on silica (0–40% 3:1 EtOAc/EtOH in cyclohexane) to afford the title compound (2.63 g, 9.25 mmol, 94% yield) as pale-yellow oil.

^{1}H NMR (400 MHz, $CDCl_3$) δ = 7.16 (d, *J* = 7.5 Hz, 1H), 6.61 (d, *J* = 7.5 Hz, 1H), 4.28–4.07 (m, 4H), 3.81–3.74 (m, 2H), 2.69 (t, *J* = 6.4 Hz, 2H), 2.36 (s, 3H), 1.93–1.85 (m, 2H), 1.27 (td, *J* = 7.1, 0.8 Hz, 6H); ^{13}C NMR (101 MHz, $CDCl_3$) δ = 154.0, 151.8 (d, *J* = 2.2 Hz), 137.4, 116.3 (d, *J* = 7.3 Hz), 115.95, 63.20 (d, *J* = 6.6 Hz), 45.9 (d, *J* = 2.2 Hz), 26.6, 23.6, 22.4 (d, *J* = 4.4 Hz), 16.0 (d, *J* = 7.3 Hz); ^{31}P{^{1}H} NMR (162 MHz, $CDCl_3$) δ = 3.0; IR (neat): 3472, 2930, 2981, 2864, 1600, 1577 cm^{-1}; LC-MS (For): 0.63 min (285) ([M+H]$^+$, 100% purity a/a); HRMS: calculated for $C_{13}H_{22}N_2O_3P^+$ ([M+H]$^+$) 285.1363, found 285.1367.

Diethyl (7-((diethoxyphosphoryl)methyl)-3,4-dihydro-1,8-naphthyridin-1(2*H*)-yl)phosphonate (7)

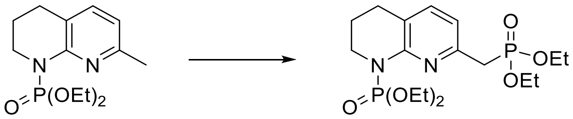

13 **7**

To a stirred solution of diethyl (7-methyl-3,4-dihydro-1,8-naphthyridin-1(2*H*)-yl)phosphonate (**13**, 4.74 g, 15.51 mmol) in THF (62 mL) under nitrogen at −42 °C, was added *s*-BuLi (1.4 M in cyclohexane, 33 mL, 46.2 mmol). The reaction was stirred for 20 min and then diethyl chlorophosphate (2.4 mL, 16.61 mmol) was added, and the reaction was stirred for 20 min. After this time, the reaction was quenched with sat. NH_4Cl (80 mL). The phases were separated and the aqueous phase was extracted with ethyl acetate (3 × 70 mL). The combined organics were passed through a hydrophobic frit and concentrated in vacuo. The residue was dissolved in a minimum volume of DMSO and purified by automated reverse-phase flash chromatography on C18 modified silica (0–60% acetonitrile in 10 mM ammonium bicarbonate solution) to afford the title compound (4.45 g, 10.59 mmol, 68% yield) as yellow oil.

^{1}H NMR (400 MHz, $CDCl_3$) δ = 7.26 (d, *J* = 7.7 Hz, 1H), 6.93 (dd, *J* = 7.7, 2.4 Hz, 1H), 4.27–4.02 (m, 8H), 3.85–3.76 (m, 2H), 3.26 (d, *J* = 22.0 Hz, 2H), 2.80–2.69 (m, 2H), 1.92 (quin, *J* = 5.7 Hz, 2H), 1.33–1.24 (m, 12H); ^{13}C NMR (101 MHz, $CDCl_3$) δ = 152.3–152.1 (m), 148.0 (d, *J* = 7.3 Hz), 137.8 (dd, *J* = 2.9, 1.5 Hz), 118.0 (dd, *J* = 7.3, 3.7 Hz), 117.1 (d, *J* = 4.4 Hz), 63.2 (d, *J* = 5.9 Hz), 62.0 (d, *J* = 6.6 Hz), 46.0 (d, *J* = 2.2 Hz), 35.8 (d, *J* = 135.7 Hz), 26.7 (s), 22.3 (d, *J* = 5.1 Hz), 16.3 (d, *J* = 5.9 Hz), 16.1 (d, *J* = 7.3 Hz); ^{31}P{^{1}H} NMR (162 MHz, $CDCl_3$) δ = 25.7, 3.0; IR (neat): 3472, 2981, 2932,

1576 cm^{-1}; LC-MS (HpH): 0.90 min (421) ([M+H]$^+$, 99% purity a/a); HRMS: calculated for $C_{17}H_{31}O_6P_2^+$ ([M+H]$^+$) 421.1652, found 421.1667.

Benzyl (*R*)-3-(2-(8-(diethoxyphosphoryl)-5,6,7,8-tetrahydro-1,8-naphthyridin-2-yl)vinyl)-3-fluoropyrrolidine-1-carboxylate (15)

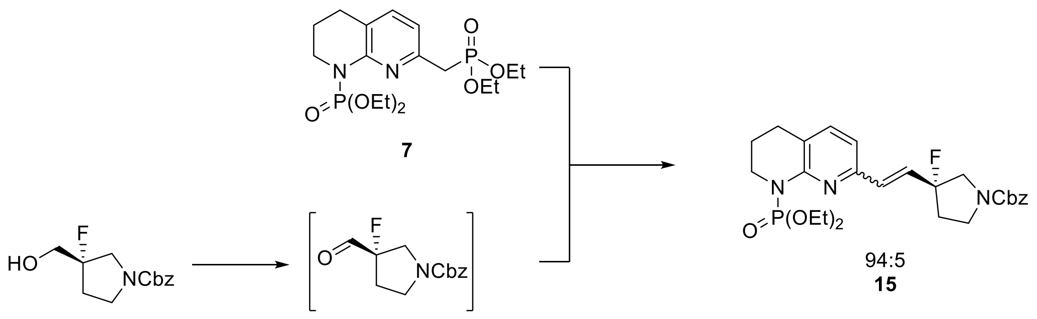

A stirred solution of benzyl (*S*)-3-fluoro-3-(hydroxymethyl)pyrrolidine-1-carboxylate (**14**, 760 mg, 3 mmol), T3P® (50% w/w in DCM, 5.73 g, 9.00 mmol) and DMSO (0.64 mL, 9.02 mmol) in DCM (10 mL) was cooled to 0 °C, and DIPEA (1.310 mL, 7.50 mmol) was added dropwise over ca. 2 min. The reaction was stirred at 0 °C for 1 h and then was washed with water (2 × 10 mL). The combined aqueous layers were back-extracted with DCM (15 mL) and the combined organics were passed through a hydrophobic frit and concentrated in vacuo.

To the residue was added a solution of diethyl ((8-(diethoxyphosphoryl)-5,6,7,8-tetrahydro-1,8-naphthyridin-2-yl)methyl)phosphonate (**7**, 509 mg, 1.211 mmol) in THF (10 mL) and the solution was cooled to 0 °C. Potassium *tert*-butoxide (1 M in THF, 7.2 mL, 7.2 mmol) was added in equal portions over 70 min until LC–MS showed complete reaction had taken place, and the reaction was quenched with sat. NH_4Cl (15 mL). The phases were separated and the aqueous layer was extracted using ethyl acetate (2 × 15 mL). The combined organics were dried using $MgSO_4$, filtered, and concentrated under a stream of nitrogen. The residue was purified by automated flash

chromatography on silica (0–40% 3:1 EtOAc/EtOH in cyclohexane) to afford the title compound as a 94:5 mixture of geometric isomers (by LC–MS), combined 584 mg (1.128 mol, 93% yield) as yellow oil.

^{1}H NMR (700 MHz, $CDCl_3$) δ = 7.41–7.28 (m, 6H), 6.95–6.84 (m, 1H), 6.73 (dd, *J* = 7.2, 4.7 Hz, 1H), 6.68 (t, *J* = 15.0 Hz, 1H), 5.22–5.10 (m, 2H), 4.25–4.15 (m, 2H), 4.13–4.03 (m, 2H), 3.88–3.71 (m, 4H), 3.70–3.61 (m, 1H), 3.60–3.50 (m, 1H), 2.77 (t, *J* = 6.4 Hz, 2H), 2.26 (td, *J* = 14.6, 6.4 Hz, 1H), 2.20–2.06 (m, 1H), 1.98–1.92 (m, 2H), 1.30–1.24 (m, 6H); ^{13}C NMR (176 MHz, $CDCl_3$) δ = 154.7 (d, *J* = 22.3 Hz), 152.0, 149.8, 137.8, 136.7 (d, *J* = 8.3 Hz), 129.9 (dd, *J* = 14.6, 10.2 Hz), 129.4 (d, *J* = 21.0 Hz), 128.4, 128.0, 127.9 (d, *J* = 9.5 Hz), 119.7 (d, *J* = 7.6 Hz), 116.2 (d, *J* = 9.5 Hz), 100.9 (dd, *J* = 179.9, 147.5 Hz), 66.9 (d, *J* = 3.2 Hz), 62.9 (d, *J* = 5.7 Hz), 56.3 (dd, *J* = 54.0, 25.4 Hz), 45.9 (d, *J* = 2.5 Hz), 44.8 (d, *J* = 61.0 Hz), 37.1 (dd, *J* = 131.6, 24.2 Hz), 27.0, 22.3 (d, *J* = 4.5 Hz), 16.2 (d, *J* = 7.0 Hz); IR (neat): 1704, 1591, 1570 cm^{-1}; LC-MS: minor: 1.23 min (518) ([M+H]$^{+}$, 5% purity a/a); major: 1.27 min (518) ([M+H]$^{+,}$ 94% purity a/a); HRMS: calculated for $C_{26}H_{34}N_3O_5P^+$ ([M+H]$^{+}$) 518.2215, found 518.2224.

*$[\alpha]_D^{20}$ not recorded as two components present

Benzyl (*S*)-3-(2-(8-(diethoxyphosphoryl)-5,6,7,8-tetrahydro-1,8-naphthyridin-2-yl)ethyl)-3-fluoropyrrolidine-1-carboxylate (16)

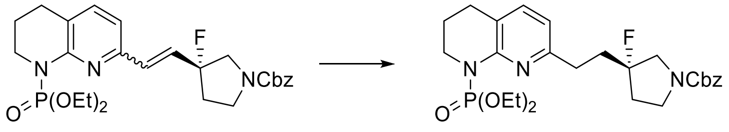

15 16

A stirred suspension of benzyl (*R*)-3-(2-(8-(diethoxyphosphoryl)-5,6,7,8-tetrahydro-1,8-naphthyridin-2-yl)vinyl)-3-fluoropyrrolidine-1-carboxylate (**15**, 522 mg, 1.009 mmol) and potassium carbonate (558 mg, 4.03 mmol) in DMF (10 mL) was heated to 100 °C, and to this, was added benzenesulfonyl hydrazide (521 mg, 3.03 mmol) portionwise over 4 min. The reaction was stirred for 30 min, cooled to rt, and partitioned between DCM (20 mL) and sat. LiCl (20 mL). The phases were separated and the aqueous phase was further extracted with DCM (2 × 20 mL). The combined organics were passed through a hydrophobic frit and co-evaporated with toluene (10 mL). The residue was purified by automated flash chromatography on silica (0–40% 3:1 EtOAc/EtOH in cyclohexane) to afford the title compound (421 mg, 0.810 mmol, 80% yield) as pale-yellow oil.

$[\alpha]_D^{20}$ (*c* = 16.7 mg/mL, MeOH): −11; ^{1}H NMR (400 MHz, $CDCl_3$) δ = 7.38–7.28 (m, 5H), 7.22 (d, *J* = 7.3 Hz, 1H), 6.66 (dd, *J* = 7.3, 1.5 Hz, 1H), 5.18–5.09 (m, 2H), 4.25–4.03 (m, 4H), 3.88–3.50 (m, 5H), 3.46–3.29 (m, 1H), 2.87–2.68 (m, 4H), 2.34–2.12 (m, 3H), 2.00–1.80 (m, 3H), 1.31–1.24 (m, 6H); ^{13}C NMR (101 MHz, $CDCl_3$) δ = 156.2, 154.7 (d, *J* = 11.7 Hz), 152.0 (d, *J* = 2.2 Hz), 137.7 (br s), 136.8 (d, *J* = 5.1 Hz), 128.4, 127.9 (d, *J* = 2.2 Hz), 127.9 (d, *J* = 5.9 Hz), 117.2 (d, *J* = 7.3 Hz), 115.6, 102.5 (dd, *J* = 176.8, 90.2 Hz), 66.8, 62.9 (d, *J* = 5.9 Hz), 55.8 (dd, *J* = 30.1, 24.9 Hz), 45.9 (d, *J* = 2.2 Hz), 44.6 (d, *J* = 33.0 Hz), 35.8 (dd, *J* = 76.3, 23.5 Hz), 35.0 (d, *J* = 22.7 Hz), 31.8 (d, *J* = 2.9 Hz), 26.7, 22.4 (d, *J* = 5.1 Hz), 16.2 (d, *J* = 7.3 Hz); IR (neat): 1702, 1599, 1577 cm^{-1}; LC-MS (HpH): 1.24 min (520) ($[M+H]^+$, 97% purity a/a); HRMS: calculated for $C_{26}H_{36}FN_3O_5P^+$ ($[M+H]^+$) 520.2371, found 520.2392.

(*S*)-7-(2-(3-Fluoropyrrolidin-3-yl)ethyl)-1,2,3,4-tetrahydro-1,8-naphthyridine (6)

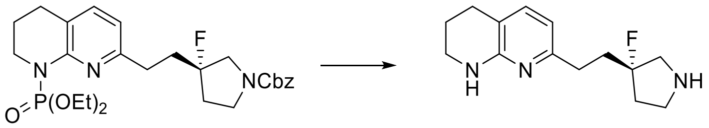

16 **6**

A stirred solution of benzyl (*S*)-3-(2-(8-(diethoxyphosphoryl)-5,6,7,8-tetrahydro-1,8-naphthyridin-2-yl)ethyl)-3-fluoropyrrolidine-1-carboxylate (**16**, 387 mg, 0.745 mmol) in 7.4 M HCl (8 mL, 59.2 mmol) was heated at 100 °C for 1.5 h. After this time, the reaction mixture was allowed to cool to rt, diluted with water (20 mL), and washed with DCM (2 × 20 mL). The aqueous phase was adjusted to pH 14 using 2 M NaOH and the organics were extracted with DCM (3 × 50 mL). The combined organics were passed through a hydrophobic frit and concentrated in vacuo to afford the title compound (160 mg, 0.642 mmol, 86% yield) as pale-brown gum.

$[\alpha]_D^{20}$ (*c* = 10.3 mg/mL, MeOH): −2; ^{1}H NMR (400 MHz, $CDCl_3$) δ = 7.02 (d, *J* = 7.3 Hz, 1H), 6.33 (d, *J* = 7.3 Hz, 1H), 4.99 (br s, 1H), 3.36 (t, *J* = 5.4 Hz, 2H), 3.20–3.07 (m, 2H), 2.94–2.84 (m, 1H), 2.79–2.55 (m, 6H), 2.19 –1.96 (m, 3H), 1.90–1.69 (m, 3H); ^{13}C NMR (101 MHz, $CDCl_3$) δ = 157.3, 155.7, 136.6, 113.3, 111.0, 105.5 (d, *J* = 174.6 Hz), 57.7 (d, *J* = 25.7 Hz), 46.0, 41.4, 37.9 (d, *J* = 22.7 Hz), 36.3 (d, *J* = 24.2 Hz), 32.6 (d, *J* = 3.7 Hz), 26.2, 21.4; IR (neat): 3254, 2928, 2844, 1597, 1586 cm^{-1}; LC-MS (HpH): 0.87 min (250) ($[M+H]^+$, 100% purity a/a); HRMS: calculated for $C_{14}H_{21}FN_3^+$ ($[M+H]^+$) 250.1714, found 250.1716.

***tert*-Butyl (*R*)-3-formylpiperidine-1-carboxylate (19)** [1]

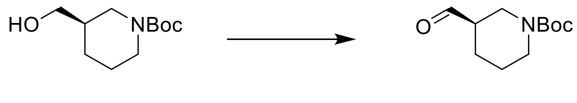

18 **19**

To a stirred solution of *tert*-butyl (*R*)-3-(hydroxymethyl)piperidine-1-carboxylate (**18**, 431 mg, 2 mmol) in bench-grade ethyl acetate (15 mL) was added IBX (1.68 g, 6.00 mmol), and the slurry stirred at 85 °C for 3 h. After this time the reaction mixture was cooled to rt, filtered under reduced pressure, and the residue was washed with ethyl acetate (2 × 10 mL). The filtrate was concentrated in vacuo to afford the title compound (455 mg) as colourless gum which was used without further purification.

7-(2-(Piperidin-3-yl)ethyl)-1,2,3,4-tetrahydro-1,8-naphthyridine (20)

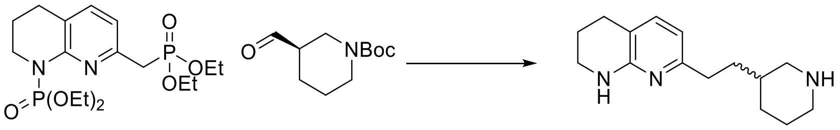

7 **19** **20**

To a stirred solution of diethyl ((8-(diethoxyphosphoryl)-5,6,7,8-tetrahydro-1,8-naphthyridin-2-yl)methyl)phosphonate (**7**, 418 mg, 0.994 mmol) and *tert*-butyl (*R*)-3-formylpiperidine-1-carboxylate (**19**, 455 mg, prepared as above) in THF (10 mL), under nitrogen at 0 °C, was added potassium *tert*-butoxide (1 M in THF, 1.79 mL, 1.79 mmol) dropwise over ca. 1 min. The reaction mixture was stirred for 20 min and then additional potassium *tert*-butoxide (1 M in THF, 0.45 mL, 0.45 mmol) was added. The reaction mixture was stirred for further 20 min and then was quenched with sat. NH_4Cl (10 mL). The phases were separated and the aqueous phase was extracted with ethyl acetate (2 × 15 mL). The combined organics were passed through a hydrophobic frit and concentrated in vacuo.

The residue was dissolved in DMF (10 mL) and the flask was charged with potassium carbonate (550 mg, 3.98 mmol). The stirred suspension was heated to 100 °C and benzenesulfonyl hydrazide (514 mg, 2.98 mmol) was added portionwise over 5 min. The reaction was stirred for 80 min and then cooled to rt. The mixture was partitioned

between DCM (50 mL) and sat. LiCl (50 mL). The phases were separated and the aqueous phase was further extracted with DCM (2 × 30 mL). The combined organics were washed with sat. LiCl (70 mL), passed through a hydrophobic frit, and concentrated in vacuo.

The residue was dissolved in 7.4 M HCl (8 mL) and heated at 100 °C for 2 h, and cooled to rt. The mixture was diluted with water (40 mL). The aqueous phase was washed with DCM (3 × 40 mL), basified with 2 M NaOH to pH ≈ 14, and extracted with DCM (8 × 50 mL). The combined organics were passed through a hydrophobic frit and concentrated in vacuo to afford the title compound (201 mg, 0.819 mmol, 82% yield) as pale-brown gum.

$[\alpha]_D^{20}$ (*c* = 10.0 mg/mL) = 0; ^{1}H NMR (700 MHz, $CDCl_3$) δ = 7.05 (d, *J* = 7.2 Hz, 1H), 6.33 (d, *J* = 7.2 Hz, 1H), 4.88 (br s, 1H), 3.42–3.38 (m, 2H), 3.26 (br d, *J* = 11.4 Hz, 1H), 3.18 (br d, *J* = 12.7 Hz, 1H), 2.68 (t, *J* = 6.4 Hz, 2H), 2.63 (td, *J* = 12.3, 2.5, 1H), 2.60–2.50 (m, 2H), 2.38 (t, *J* = 11.7 Hz, 1H), 1.94–1.88 (m, 3H), 1.78–1.69 (m, 2H), 1.69–1.51 (m, 4H), 1.14–1.06 (m, 1H); ^{13}C NMR (176 MHz, $CDCl_3$) δ = 158.0, 155.7, 136.7, 113.3, 111.2, 51.3, 45.9, 41.6, 35.4, 34.7, 34.3, 30.5, 26.4, 24.8, 21.5; IR (neat): 3254, 2924, 2844, 1599 cm^{-1}; LC-MS (HpH): 0.90 min (246) ([M+H]$^+$, 95% purity a/a); HRMS: calculated for $C_{15}H_{24}N_3^+$ ([M+H]$^+$) 246.1965, found 246.1961.

tert-Butyl (_R_)-3-formylpyrrolidine-1-carboxylate (22) [2]

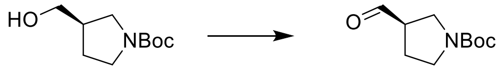

21 **22**

To a stirred solution of *tert*-butyl (*R*)-3-(hydroxymethyl)pyrrolidine-1-carboxylate (**21**, 0.207 g, 1.028 mmol) in bench-grade ethyl acetate (7 mL) was added IBX (0.864 g, 3.09 mmol) and the slurry was stirred at 85 °C for 2.25 h. After this time, the reaction

mixture was cooled to rt, filtered under reduced pressure, and the residue was washed with ethyl acetate (2 × 10 mL). The filtrate was concentrated in vacuo to afford the title compound (216 mg) as colourless gum which was used without further purification.

7-(2-(Pyrrolidin-3-yl)ethyl)-1,2,3,4-tetrahydro-1,8-naphthyridine (23) [3]

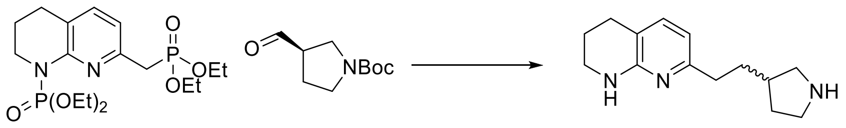

7 **22** **23**

To a stirred solution of diethyl ((8-(diethoxyphosphoryl)-5,6,7,8-tetrahydro-1,8-naphthyridin-2-yl)methyl)phosphonate (**7**, 210 mg, 0.50 mmol) and *tert*-butyl (*R*)-3-formylpyrrolidine-1-carboxylate (**22**, 216 mg, prepared as above) in THF (10 mL), under nitrogen at 0 °C, was added potassium *tert*-butoxide (1 M in THF, 0.75 mL, 0.750 mmol) dropwise over ca. 1 min. The reaction mixture was stirred for 7 min and then quenched by the addition of NH_4Cl (10 mL). The phases were separated and the aqueous phase was extracted with ethyl acetate (2 × 10 mL). The combined organics were passed through a hydrophobic frit and concentrated under a flow of nitrogen.

The residue was dissolved in DMF (10 mL) and the flask was charged with potassium carbonate (276 mg, 2.0 mmol). The stirred suspension was heated to 100 °C and benzenesulfonyl hydrazide (258 mg, 1.5 mmol) was added portionwise over 4 min. After 10 min additional potassium carbonate (276 mg, 2.0 mmol) was added followed by benzenesulfonyl hydrazide (258 mg, 1.5 mmol) portionwise over 4 min. After 1 h, the reaction mixture was cooled to rt and partitioned between DCM (20 mL) and sat. LiCl (20 mL). The phases were separated and the aqueous phase was further extracted with DCM (2 × 30 mL). The combined organics were washed with sat. LiCl (70 mL), passed through a hydrophobic frit, and concentrated in vacuo.

The residue was dissolved in 7.4 M HCl (8 mL), the solution heated at 100 °C for 2 h, and cooled to rt. The mixture was diluted with water (20 mL). The aqueous phase was washed with DCM (2 × 20 mL), basified with 2 M NaOH to pH ≈ 14, and extracted with DCM (3 × 70 mL). The combined organics were passed through a hydrophobic frit and concentrated in vacuo to afford the title compound (96 mg, 0.415 mmol, 83% yield) as the (*R*)-enantiomer as brown gum in 30% ee.

^{1}H NMR (400 MHz, $CDCl_3$)* δ = 7.04 (d, *J* = 7.3 Hz, 1H), 6.33 (d, *J* = 7.3 Hz, 1H), 4.81 (br s, 1H), 3.38 (t, *J* = 5.4 Hz, 2H), 3.08 (dd, *J* = 10.8, 7.4 Hz, 1H), 2.98–2.83 (m, 2H), 2.67 (t, *J* = 6.4 Hz, 2H), 2.54 (td, *J* = 8.1, 2.9 Hz, 2H), 2.48 (dd, *J* = 11.0, 7.6 Hz, 1H), 2.37 (br s, 1H), 2.09–1.85 (m, 4H), 1.70 (q, *J* = 7.8 Hz, 2H), 1.35 (dq, *J* = 12.2, 7.7 Hz, 1H); ^{13}C NMR (101 MHz, $CDCl_3$) δ = 158.3, 155.7, 136.6, 113.1, 111.1, 53.1, 46.8, 41.6, 39.5, 37.0, 34.7, 32.6, 26.3, 21.5; IR (neat): 3253, 2926, 2855, 1599 cm^{-1}; LC-MS (HpH): 0.75 min (232) ([M+H]$^+$, 100% purity a/a); HRMS: calculated for $C_{14}H_{22}N_3^+$ ([M+H]$^+$) 232.1808, found 232.1816.

*A sample of analytical purity was obtained for ^{1}H NMR spectroscopy and chiral HPLC analysis by mass-directed HPLC

7-(2-(Azetidin-3-yl)ethyl)-1,2,3,4-tetrahydro-1,8-naphthyridine (25) [4]

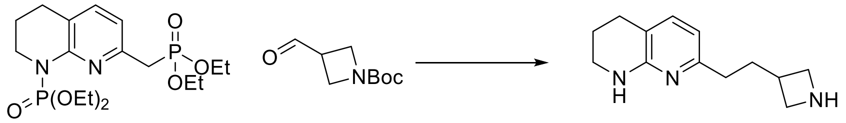

7 24 25

To a stirred solution of diethyl ((8-(diethoxyphosphoryl)-5,6,7,8-tetrahydro-1,8-naphthyridin-2-yl)methyl)phosphonate (**7**, 417 mg, 0.992 mmol) and *tert*-butyl 3-formylazetidine-1-carboxylate (**24**, 276 mg, 1.488 mmol) in THF (8 mL) under nitrogen at 0 °C, was added potassium *tert*-butoxide (1 M in THF, 1.8 mL, 1.8 mmol) dropwise over ca. 1 min. The reaction mixture was stirred for 5 min and then quenched with sat.

NH_4Cl (10 mL). The phases were separated and the organic phase was extracted with ethyl acetate (2 × 15 mL). The combined organics were passed through a hydrophobic frit and concentrated in vacuo.

The residue was dissolved in DMF (10 mL) and the flask was charged with potassium carbonate (548 mg, 3.97 mmol). The stirred suspension was heated to 100 °C and benzenesulfonyl hydrazide (512 mg, 2.98 mmol) was added portionwise over 5 min. The reaction mixture was stirred for 75 min and then cooled to rt. The mixture was partitioned between DCM (50 mL) and sat. LiCl (50 mL). The phases were separated and the aqueous phase was extracted with DCM (3 × 40 mL). The combined organics were washed with sat. LiCl (70 mL), passed through a hydrophobic frit, and concentrated in vacuo.

The residue was dissolved in 7.4 M HCl (9 mL) and stirred at 100 °C for 3.5 h. The reaction mixture then was stirred at rt for another 1 h, and diluted with water (40 mL). The aqueous phase was washed with DCM (2 × 40 mL), basified with 2 M NaOH to pH ≈ 14, and extracted with DCM (5 × 100 mL). The combined organics were passed through a hydrophobic frit and concentrated in vacuo to afford the title compound (171 mg, 0.787 mmol, 79% yield) as brown gum.

^1H NMR (400 MHz, $CDCl_3$) δ = 7.03 (d, *J* = 7.3 Hz, 1H), 6.30 (d, *J* = 7.3 Hz, 1H), 4.87 (br s, 1H), 3.63 (t, *J* = 8.1 Hz, 2H), 3.45–3.33 (m, 4H), 3.01 (br s, 1H), 2.84–2.71 (m, 1H), 2.68 (t, *J* = 6.3 Hz, 2H), 2.48–2.40 (m, 2H), 1.95–1.86 (m, 4H); ^{13}C NMR (101 MHz, $CDCl_3$) δ = 157.8, 155.7, 136.5, 113.2, 111.2, 53.2, 41.6, 35.4, 35.0, 34.6, 26.3, 21.5; IR (neat): 3352, 2938, 2844, 1598 cm^{-1}; LC-MS (HpH): 0.67 min (218) ($[M+H]^+$, 100% purity a/a); HRMS: calculated for $C_{13}H_{20}N_3^+$ ($[M+H]^+$) 218.1652, found 218.1648.

7-(3-(Azetidin-3-yl)propyl)-1,2,3,4-tetrahydro-1,8-naphthyridine (27)

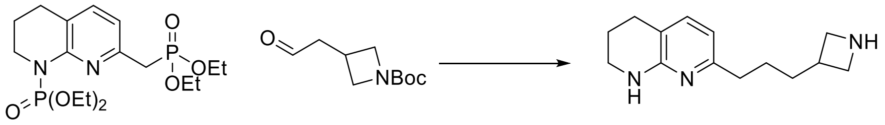

7 **26** **27**

To a stirred solution of diethyl ((8-(diethoxyphosphoryl)-5,6,7,8-tetrahydro-1,8-naphthyridin-2-yl)methyl)phosphonate (**7**, 455 mg, 1.082 mmol) at 0 °C was added sodium hydride (60% dispersion in mineral oil, 51.9 mg, 1.299 mmol). After 5 min, *tert*-butyl 3-(2-oxoethyl)azetidine-1-carboxylate (**26**, 237 mg, 1.191 mmol) was added in one portion. After 15 min, a further 1.2 equiv of sodium hydride (60% dispersion in mineral oil, 51.9 mg, 1.299 mmol) was added. After stirring for 50 min, the reaction was quenched with sat. NH_4Cl (10 mL). The phases were separated and the organic phase was extracted with ethyl acetate (2 × 15 mL). The combined organics were passed through a hydrophobic frit and concentrated in vacuo.

The residue was dissolved in DMF (10 mL), heated to 100 °C, and potassium carbonate (599 mg, 4.33 mmol) and benzenesulfonyl hydrazide (559 mg, 3.25 mmol) were added portionwise over 5 min. After 2 h, the reaction mixture was cooled to rt and partitioned between DCM (25 mL) and sat. LiCl solution. The aqueous phase was extracted with DCM (3 × 30 mL), the combined organics washed with sat. LiCl solution (80 mL), passed through a hydrophobic frit, and concentrated in vacuo.

The residue was dissolved in 7.4 M HCl (5 mL) and stirred at 100 °C for 2 h. The reaction mixture was stirred at rt for another 2 h, and diluted with water (40 mL). The aqueous phase was washed with DCM (2 × 40 mL), basified with 2 M NaOH to pH ≈ 14, and extracted with DCM (5 × 50 mL). The combined organics were dried over Na_2SO_4, filtered, and concentrated in vacuo to afford the title compound (158 mg, 0.683 mmol, 63% yield) as pale-brown gum.

^{1}H NMR (400 MHz, $CDCl_3$)* δ = 7.05 (d, *J* = 7.3 Hz, 1H), 6.33 (d, *J* = 7.3 Hz, 1H), 4.81 (br s, 1H), 3.67 (t, *J* = 7.6 Hz, 2H), 3.45–3.32 (m, 4H), 2.79–2.65 (m, 3H), 2.52 (t, *J* = 7.3 Hz, 2H), 1.95–1.86 (m, 2H), 1.68–1.50 (m, 4H); ^{13}C NMR (101 MHz, $CDCl_3$) δ = 158.0, 155.7, 136.6, 113.2, 111.2, 53.1, 41.6, 37.7, 35.1, 34.2, 27.3, 26.3, 21.5; IR (neat): 3262, 2937, 1599 cm^{-1}; LC-MS (HpH): 0.85 min (232) ([M+H]$^+$, 98% purity a/a); HRMS: calculated for $C_{14}H_{22}N_3^+$ ([M+H]$^+$) 232.1808, found 232.1812.

*One exchangeable ^{1}H not observed

Diethyl (*E*)-(7-(2-(8-(diethoxyphosphoryl)-5,6,7,8-tetrahydro-1,8-naphthyridin-2-yl)vinyl)-3,4-dihydro-1,8-naphthyridin-1(2*H*)-yl)phosphonate (28)

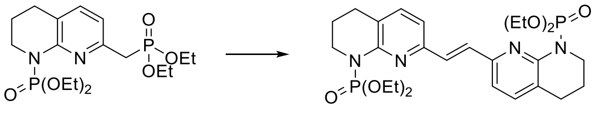

7 **28**

To a stirred solution of diethyl ((8-(diethoxyphosphoryl)-5,6,7,8-tetrahydro-1,8-naphthyridin-2-yl)methyl)phosphonate (**7**, 75 mg, 0.178 mmol) in THF (2 mL) was added potassium *tert*-butoxide (1 M in THF, 0.21 mL, 0.210 mmol). The reaction mixture was stirred for 1 h, then diluted with ethyl acetate (10 mL), and quenched with sat. NH_4Cl (15 mL). The phases were separated and the aqueous phase was extracted with ethyl acetate (2 × 20 mL). The combined organics were passed through a hydrophobic frit and concentrated in vacuo to afford the title compound (34 mg, 0.060 mmol, 68% yield) as yellow oil.

^{1}H NMR (700 MHz, $CDCl_3$) δ = 7.57 (s, 2H), 7.29 (d, *J* = 7.6 Hz, 2H), 6.85 (d, *J* = 7.6 Hz, 2H), 4.29–4.21 (m, 4H), 4.18–4.11 (m, 4H), 3.89–3.84 (m, 4H), 2.79 (t, *J* = 6.1 Hz, 4H), 1.96 (quin, *J* = 5.5 Hz, 4H), 1.30 (t, *J* = 7.0 Hz, 12H); ^{13}C NMR (176 MHz, $CDCl_3$) δ = 152.1, 151.5, 137.7, 131.1, 119.3 (d, *J* = 7.3 Hz), 116.7, 63.1 (d, *J* = 6.0 Hz), 46.0 (d, *J* = 2.2 Hz), 27.1, 22.4 (d, *J* = 4.8 Hz), 16.2 (d, *J* = 7.3 Hz); IR (neat): 2984, 2939,

1573, 1456, 1409 cm^{-1}; LC-MS (HpH): 1.17 min (565) ([M+H]$^+$, 95% purity a/a); HRMS: calculated for $C_{26}H_{39}N_4O_6P_2^+$ ([M+H]$^+$), 565.2339, found 565.2346.

The ^{13}C satellites of the alkene present as a 15 Hz doublet, indicative of the *trans* alkene geometry

***tert*-Butyl (*R*)-3-(2-(8-(diethoxyphosphoryl)-5,6,7,8-tetrahydro-1,8-naphthyridin-2-yl)ethyl)pyrrolidine-1-carboxylate (30), diethyl (7-(hydroxymethyl)-3,4-dihydro-1,8-naphthyridin-1(2*H*)-yl)phosphonate (31), and tetraethyl (ethane-1,2-diylbis(3,4-dihydro-1,8-naphthyridine-7,1(2*H*)-diyl))bis(phosphonate) (32)**

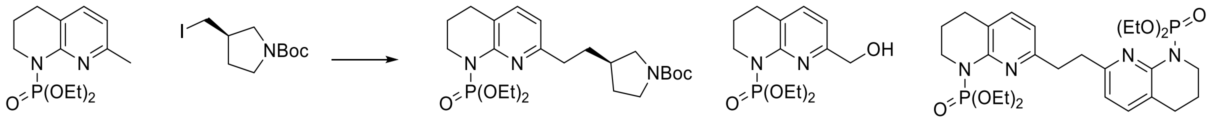

13 **29** **30** **31** **32**

To a stirred solution of diethyl (7-methyl-3,4-dihydro-1,8-naphthyridin-1(2*H*)-yl)phosphonate (**13**, 284 mg, 1 mmol) in THF (5 mL), at −78 °C under nitrogen, was added *sec*-butyllithium (1.1 M in cyclohexane, 1.35 mL, 1.485 mmol). The reaction mixture was stirred for 20 min and then a solution of *tert*-butyl (*R*)-3-(iodomethyl)pyrrolidine-1-carboxylate (**29**, 405 mg, 1.300 mmol) in THF (5 mL) was added. The reaction mixture was stirred for 10 min, quenched by the addition of sat. NH_4Cl (10 mL), and extracted with ethyl acetate (3 × 10 mL). The combined organics were dried over Na_2SO_4 and concentrated in vacuo. The residue was purified by automated flash chromatography on silica (0–70% 3:1 EtOAc/EtOH) to afford *tert*-butyl (*R*)-3-(2-(8-(diethoxyphosphoryl)-5,6,7,8-tetrahydro-1,8-naphthyridin-2-yl)ethyl)pyrrolidine-1-carboxylate (**30**, 99 mg, 0.212 mmol, 21% yield) as pale yellow oil. $[\alpha]_D^{20}$ (*c* = 10.0 mg/mL, MeOH): −4; ^{1}H NMR (400 MHz, $CDCl_3$) δ = 7.20 (d, *J* = 7.7 Hz, 1H), 6.63 (d, *J* = 7.7 Hz, 1H), 4.28–3.99 (m, 4H), 3.88–3.77 (m, 2H), 3.64–3.34

(m, 2H), 3.29–3.15 (m, 1H), 2.95–2.80 (m, 1H), 2.73 (t, *J* = 6.4 Hz, 2H), 2.64 (t, *J* = 7.6 Hz, 2H), 2.21–1.96 (m, 2H), 1.92 (quin, *J* = 5.9 Hz, 2H), 1.86–1.75 (m, 2H), 1.58–1.41 (m, 10H), 1.28 (t, *J* = 7.1 Hz, 6H); ^{13}C NMR (101 MHz, $CDCl_3$) δ = 157.3, 154.5, 151.9 (d, *J* = 1.5 Hz), 137.5, 116.9 (d, *J* = 7.3 Hz), 115.5, 78.9, 62.9 (d, *J* = 5.9 Hz), 51.4 (d, *J* = 45.5 Hz), 46.0 (d, *J* = 2.9 Hz), 45.6 (d, *J* = 33.0 Hz), 38.1 (d, *J* = 82.9 Hz), 36.0, 32.8, 31.4 (d, *J* = 76.3 Hz), 28.5, 26.8 (3C), 22.4 (d, *J* = 4.4 Hz), 16.2 (d, *J* = 7.3 Hz); IR (neat): 2976, 2931, 2866, 1693, 1576, 1466, 1409 cm^{-1}; LC-MS (HpH): 1.32 min (468) ([M+H]$^+$, 100% purity a/a); HRMS: calculated for $C_{23}H_{39}N_3O_5P^+$ ([M+H]$^+$) 468.2622, found 468.2639.

Diethyl (7-(hydroxymethyl)-3,4-dihydro-1,8-naphthyridin-1(*2H*)-yl)phosphonate (**31**, 61 mg, 0.203 mmol, 20% yield) as yellow oil. ^{1}H NMR (600 MHz, $CDCl_3$) δ = 7.32 (d, *J* = 7.7 Hz, 1H), 6.69 (d, *J* = 7.7 Hz, 1H), 4.61 (s, 2H), 4.30 (br s, 1H), 4.22–4.14 (m, 2H), 4.11–4.03 (m, 2H), 3.89–3.83 (m, 2H), 2.79 (t, *J* = 6.4 Hz, 2H), 1.96 (quin, *J* = 5.9 Hz, 2H), 1.29 (t, *J* = 7.2 Hz, 6H); ^{13}C NMR (151 MHz, $CDCl_3$) δ = 154.6, 151.1 (d, *J* = 1.1 Hz), 138.2, 117.9 (d, *J* = 7.2 Hz), 112.7, 62.9, 62.9 (d, *J* = 6.1 Hz), 45.9 (d, *J* = 2.8 Hz), 26.7 (d, *J* = 1.1 Hz), 22.2 (d, *J* = 4.4 Hz), 16.1 (d, *J* = 7.2 Hz); IR (neat): 3378, 2982, 2932, 1579, 1466, 1410 cm^{-1}; LC-MS (HpH): 0.80 min (301) ([M+H]$^+$, 98% purity a/a); HRMS: calculated for $C_{13}H_{22}N_2O_4P$ ([M+H]$^+$) 301.1312, found 301.1315.

Tetraethyl (ethane-1,2-diylbis(3,4-dihydro-1,8-naphthyridine-7,1(2*H*)-diyl))bis(phosphonate) (**32**, 28 mg, 0.049 mmol, 5% yield) as off-white amorphous solid. Mp: dec >130 °C; ^{1}H NMR (600 MHz, $CDCl_3$) δ = 7.20 (d, *J* = 7.5 Hz, 2H), 6.63 (d, *J* = 7.5 Hz, 2H), 4.27–4.18 (m, 4H), 4.17–4.07 (m, 4H), 3.86–3.82 (m, 4H), 3.05 (s, 4H), 2.74 (t, *J* = 6.3 Hz, 4H), 1.93 (quin, *J* = 5.8 Hz, 4H), 1.30 (td, *J* = 7.1, 0.6 Hz, 12H); ^{13}C NMR (151 MHz, $CDCl_3$) δ = 157.4, 151.9 (d, *J* = 1.9 Hz), 137.5, 116.9 (d, *J* = 7.2

Hz), 115.6, 63.0 (d, J = 5.8 Hz), 46.0 (d, J = 2.5 Hz), 37.1, 26.8, 22.5 (d, J = 4.4 Hz), 16.2 (d, J = 7.2 Hz); IR (neat): 2981, 2935, 2868, 1575, 1465, 1410 cm^{-1}; LC-MS (HpH): 1.19 min (567) ($[M+H]^+$, 93% purity a/a); HRMS: calculated for $C_{26}H_{41}N_4O_6P_2^+$ ($[M+H]^+$) 567.2496, found 567.2506.

(*R*)-7-(2-(Pyrrolidin-3-yl)ethyl)-1,2,3,4-tetrahydro-1,8-naphthyridine ((*R*)-23)

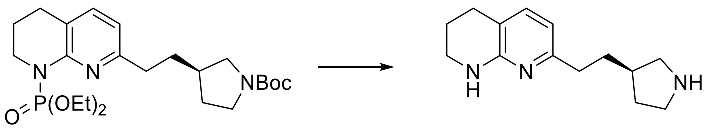

30 **(*R*)-23**

A stirred solution of *tert*-butyl (*R*)-3-(2-(8-(diethoxyphosphoryl)-5,6,7,8-tetrahydro-1,8-naphthyridin-2-yl)ethyl)pyrrolidine-1-carboxylate (**30**, 103 mg, 0.221 mmol) in 7.4 M HCl (5 mL, 37.0 mmol) was heated at 100 °C for 3.5 h. After this time, the reaction was allowed to cool to rt, diluted with water (10 mL), and washed with DCM (2 × 15 mL). The aqueous phase was adjusted to pH ≈ 14 using 2 M NaOH and the organics were extracted with 10:1 $CHCl_3$/IPA (5 × 20 mL). The combined organics were passed through a hydrophobic frit and concentrated in vacuo to afford (*R*)-7-(2-(pyrrolidin-3-yl)ethyl)-1,2,3,4-tetrahydro-1,8-naphthyridine ((*R*)-**23**, 47 mg, 0.203 mmol, 92% yield) as brown gum in 99% ee.

$[\alpha]_D^{20}$ (c = 10.0 mg/mL): −4. Remaining analytical data are consistent with racemised compound **23**.

References

1. Peschke, B.; Ankersen, M.; Bauer, M.; Hansen, T. K.; Hansen, B. S.; Nielsen, K. K.; Raun, K.; Richter, L.; Westergaard, L. *Eur. J. Med. Chem.* **2002**, *37* (6), 487-501.
2. Horiuchi, T.; Ohta, T.; Shirakawa, E.; Nozaki, K.; Takaya, H. *J. Org. Chem.* **1997**, *62* (13), 4285-4292.
3. Procopiou, P. A.; Anderson, N. A.; Barrett, J.; Barrett, T. N.; Crawford, M. H. J.; Fallon, B. J.; Hancock, A. P.; Le, J.; Lemma, S.; Marshall, R. P.; Morrell, J.; Pritchard, J. M.; Rowedder, J. E.; Saklatvala, P.; Slack, R. J.; Sollis, S. L.; Suckling, C. J.; Thorp, L. R.; Vitulli, G.; Macdonald, S. J. F. *J. Med. Chem.* **2018**, *61* (18), 8417-8443.
4. Devasthale, P.; Moore, F.; Zhao, G.; Pieniazek, S. N.; Selvakumar, K.; Dhanusu, S.; Panda, M.; Marcin, L. R. CYCLOBUTANE- AND AZETIDINE-CONTAINING MONO AND SPIROCYCLIC COMPOUNDS AS ALPHA V INTEGRIN INHIBITORS. Pat. WO2018089355, 17 May 2018.

^{1}H and ^{13}C NMR spectra of compounds

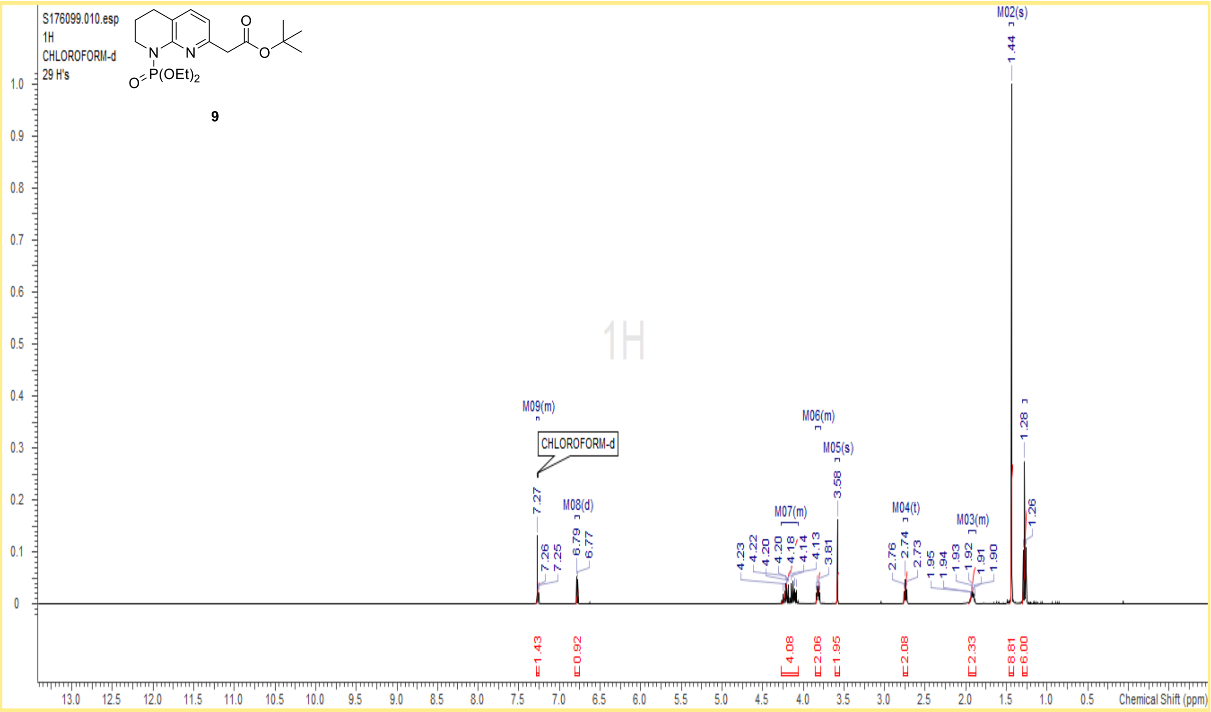

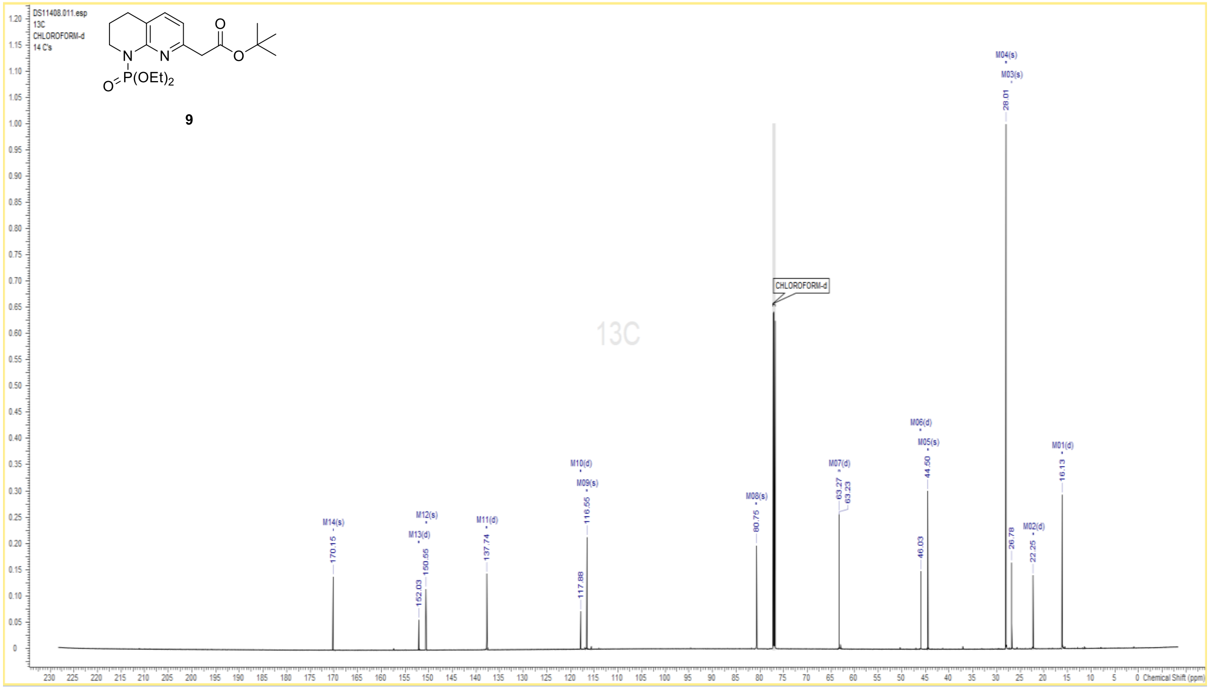

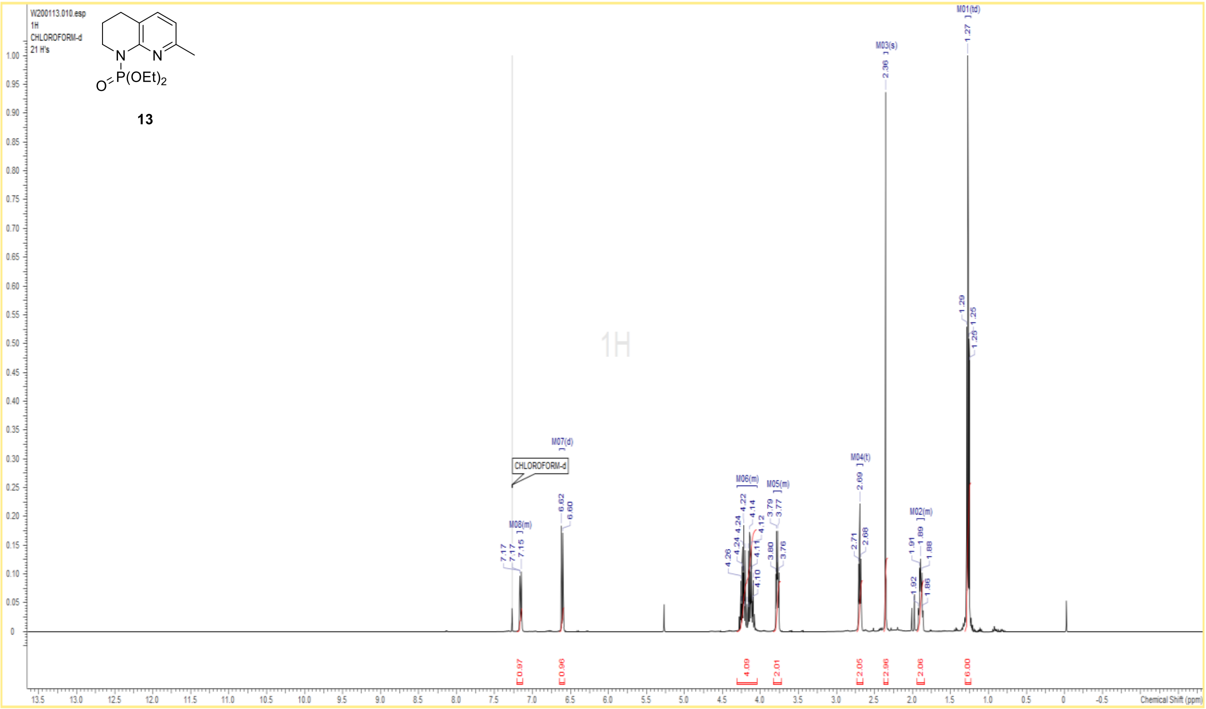

W200113.010.esp
1H
CHLOROFORM-d
21 H's
N
N
P(OEt)2
13
1H
CHLOROFORM-d
M01(td)
M02(m)
M03(s)
M04(t)
M05(m)
M06(m)
M07(d)
M08(m)
Chemical Shift (ppm)

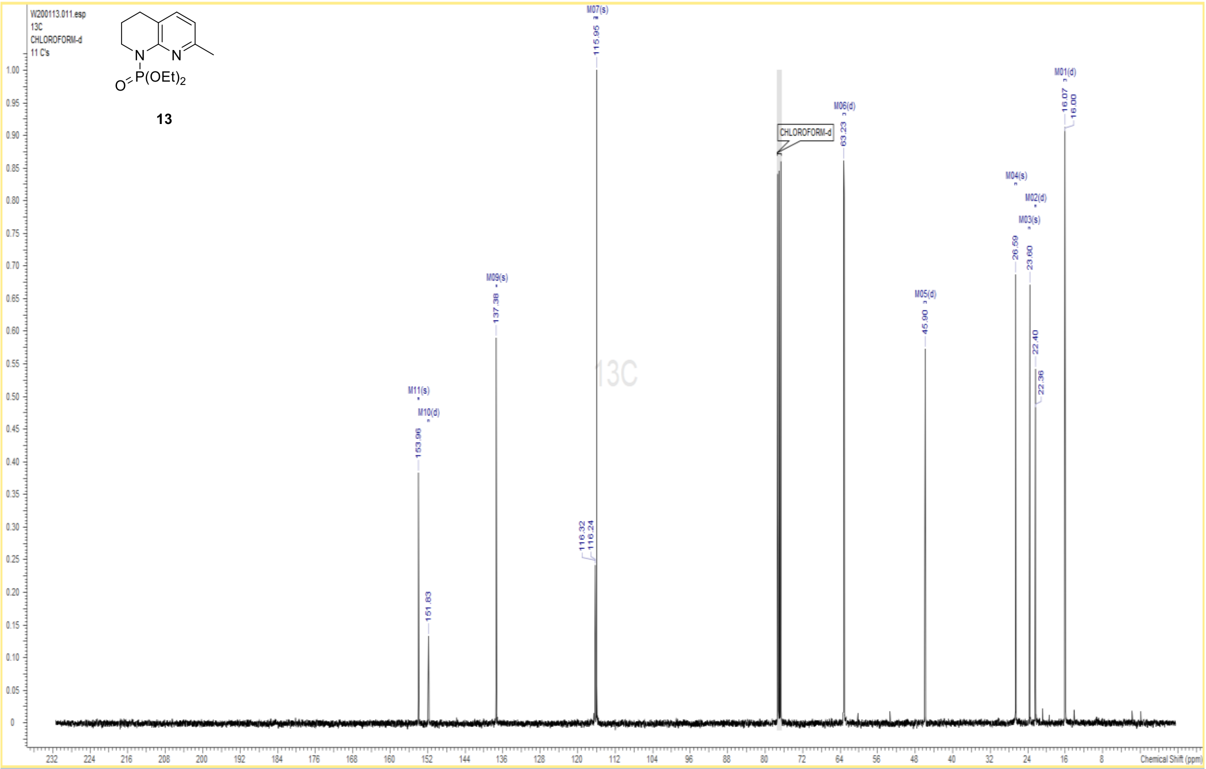

W200113.011.esp
13C
CHLOROFORM-d
11 C's
N
N
P(OEt)2
13
13C
CHLOROFORM-d
Chemical Shift (ppm)

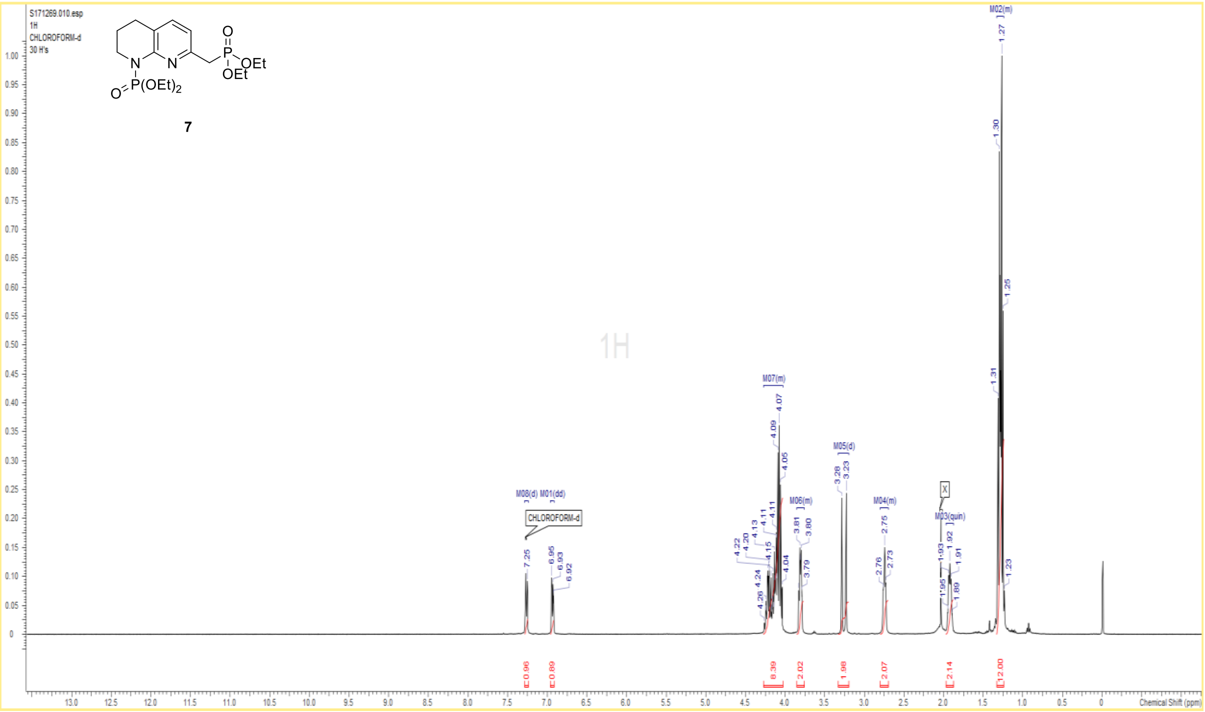
S171269.010.esp
1H
CHLOROFORM-d
30 H's
7
1H
CHLOROFORM-d
Chemical Shift (ppm)

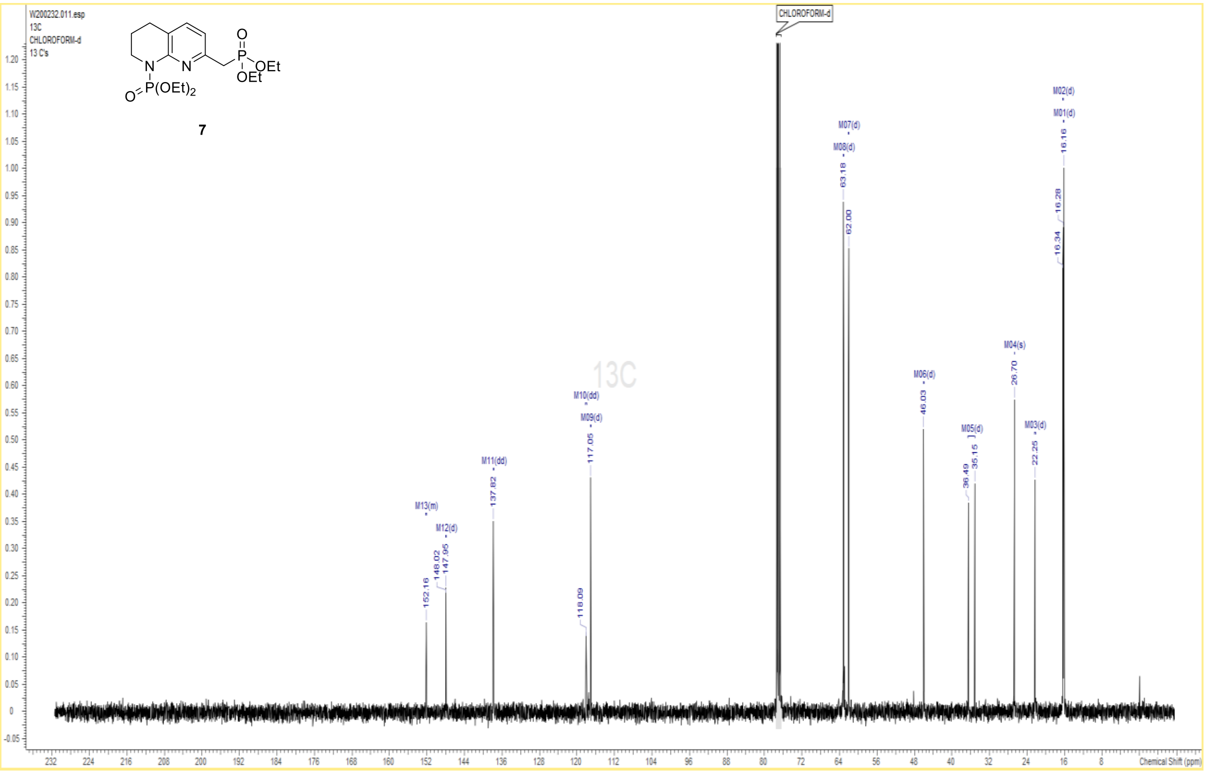
W200232.011.esp
13C
CHLOROFORM-d
13 C's
7
13C
CHLOROFORM-d
Chemical Shift (ppm)

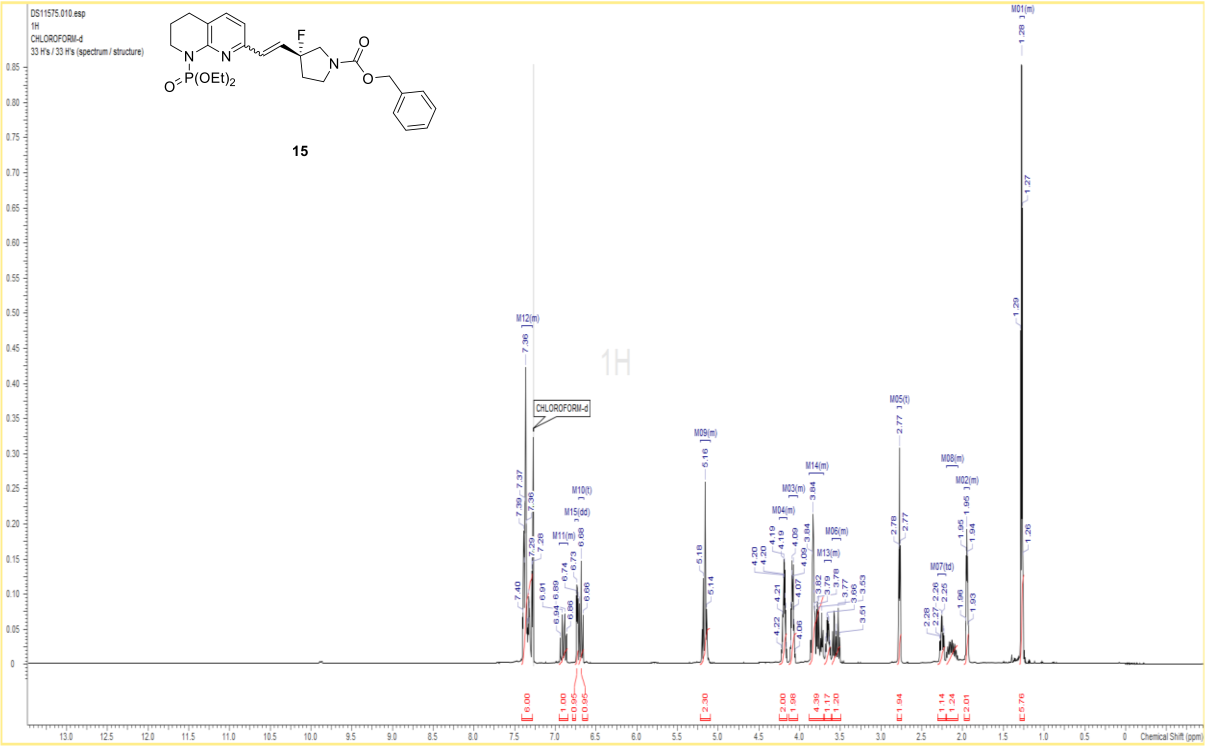

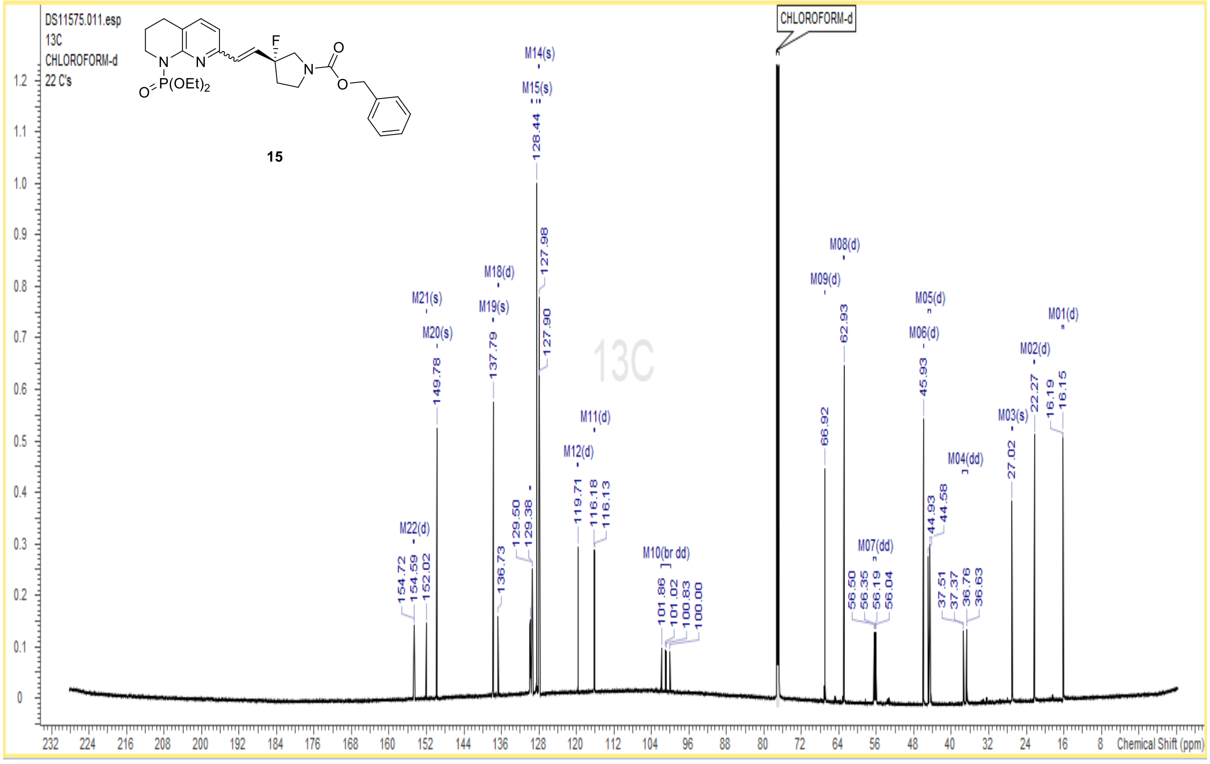

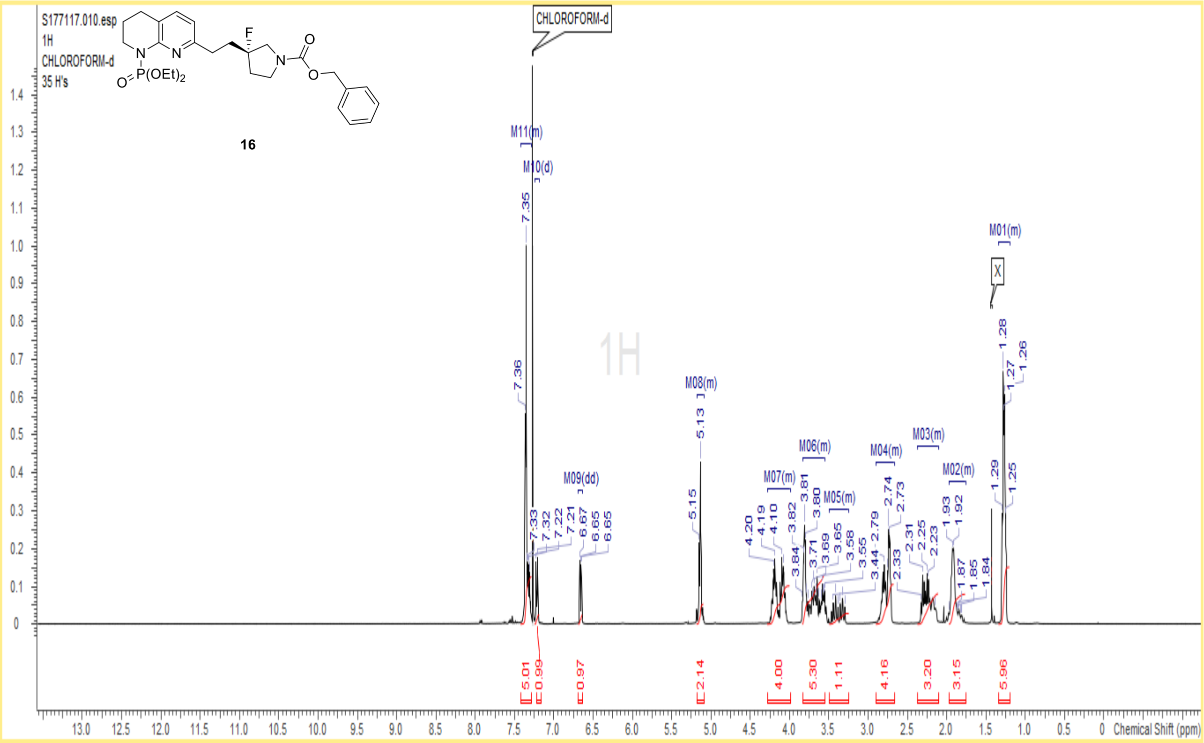
S177117.010.esp
1H
CHLOROFORM-d
35 H's
16
CHLOROFORM-d
1H
Chemical Shift (ppm)

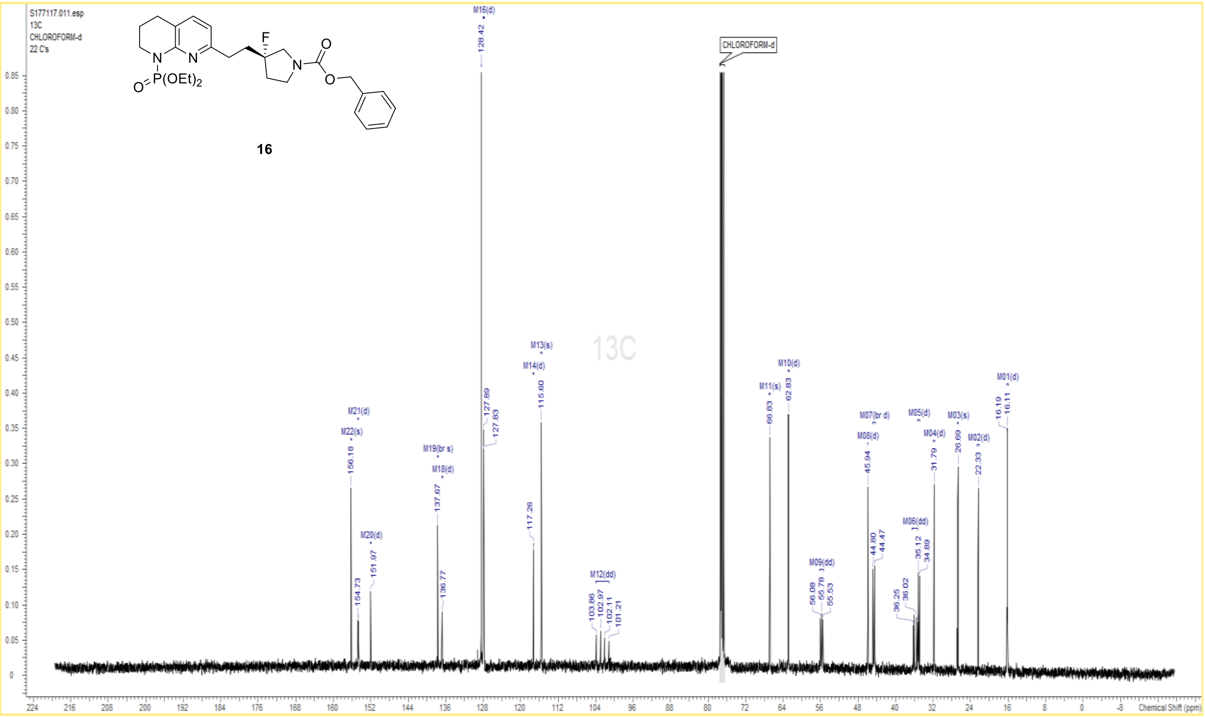
S177117.011.esp
13C
CHLOROFORM-d
22 C's
16
CHLOROFORM-d
13C
Chemical Shift (ppm)

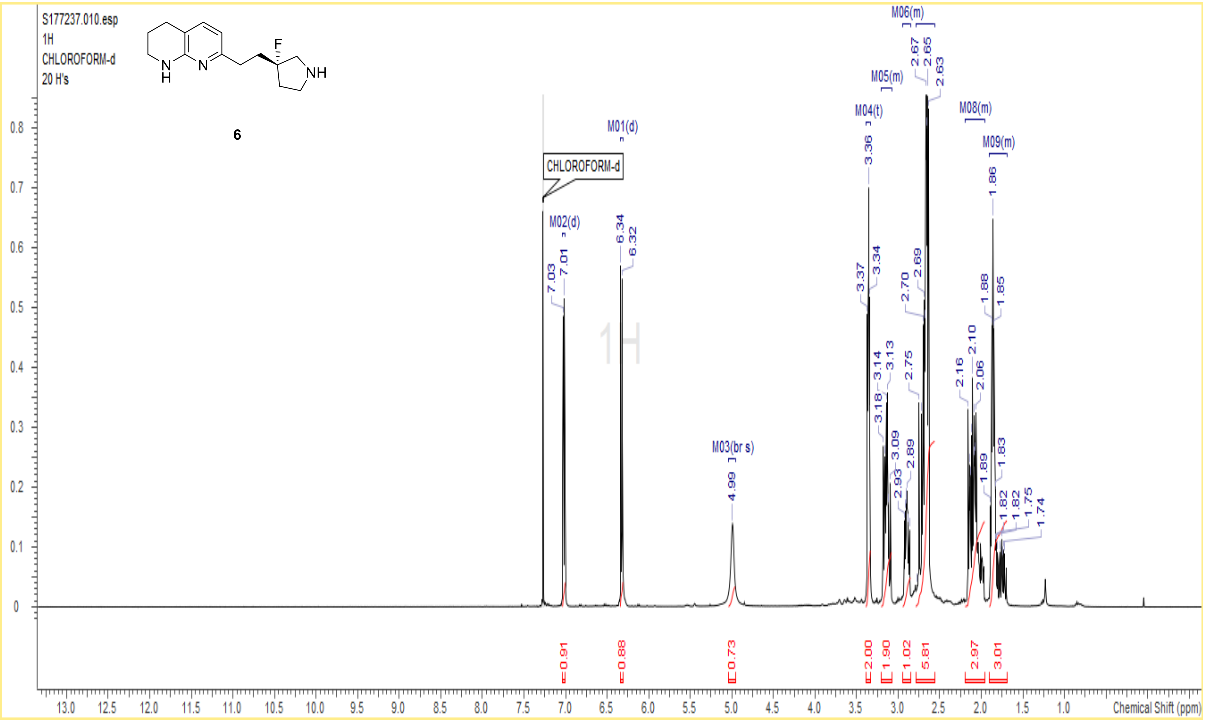
S177237.010.esp
1H
CHLOROFORM-d
20 H's
6
CHLOROFORM-d
M01(d)
M02(d)
M03(br s)
M04(t)
M05(m)
M06(m)
M08(m)
M09(m)
Chemical Shift (ppm)

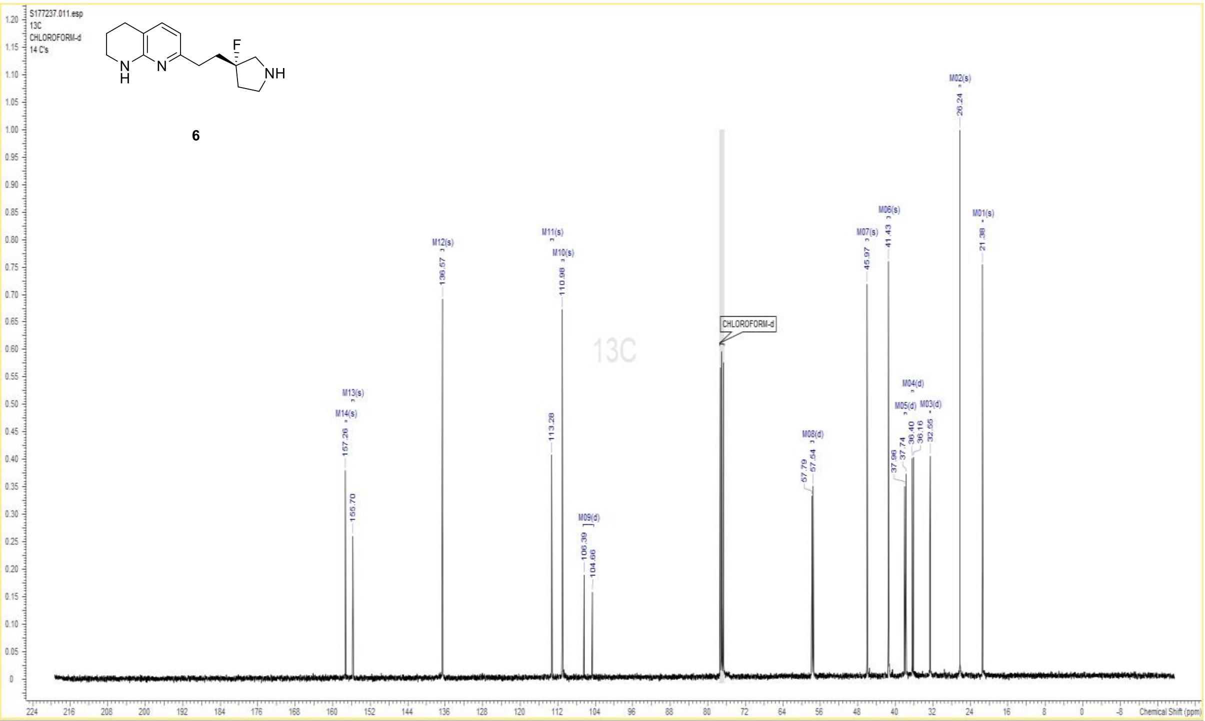
S177237.011.esp
13C
CHLOROFORM-d
14 C's
6
CHLOROFORM-d
Chemical Shift (ppm)

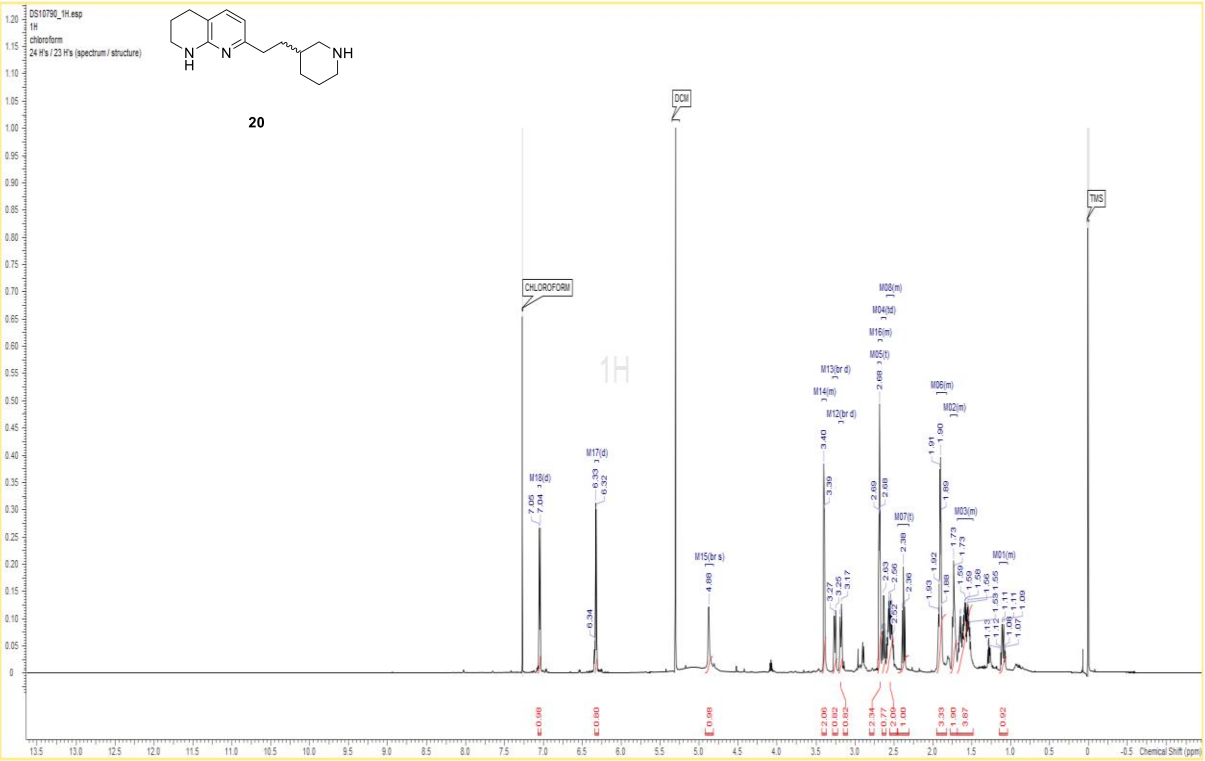
DS10790_1H.esp
1H
chloroform
24 H's / 23 H's (spectrum / structure)
20
CHLOROFORM
DCM
TMS
1H
Chemical Shift (ppm)

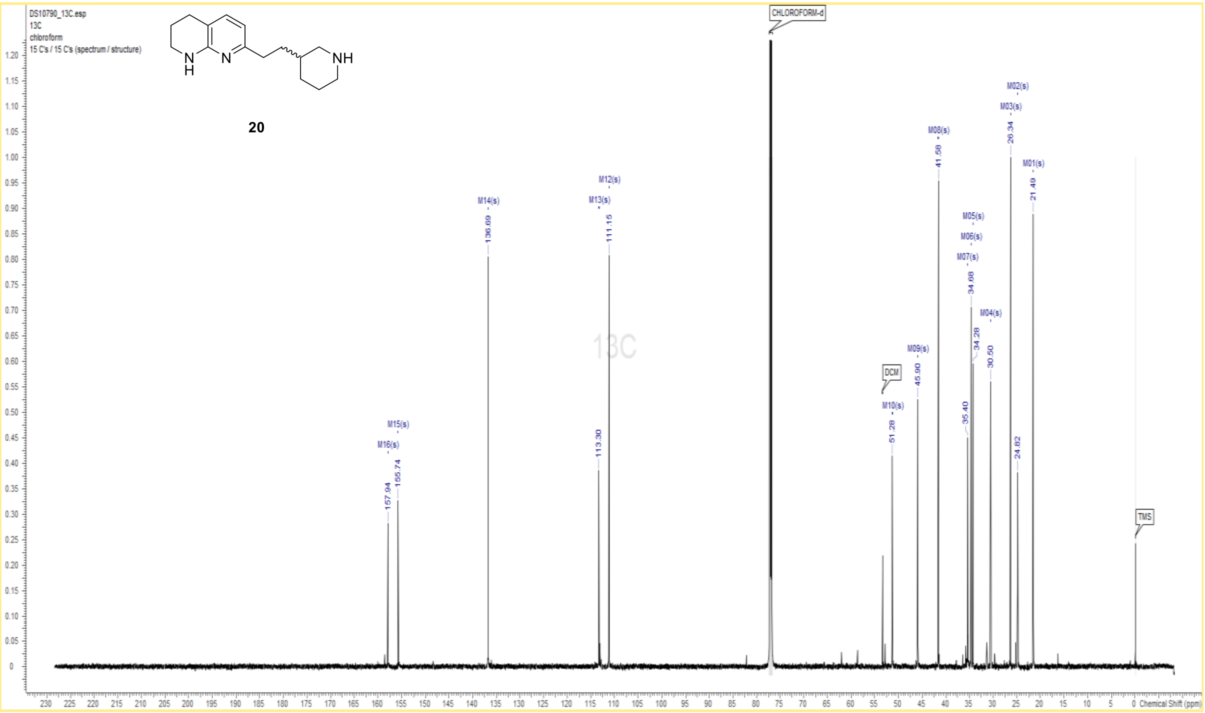
DS10790_13C.esp
13C
chloroform
15 C's / 15 C's (spectrum / structure)
20
CHLOROFORM-d
DCM
TMS
13C
Chemical Shift (ppm)

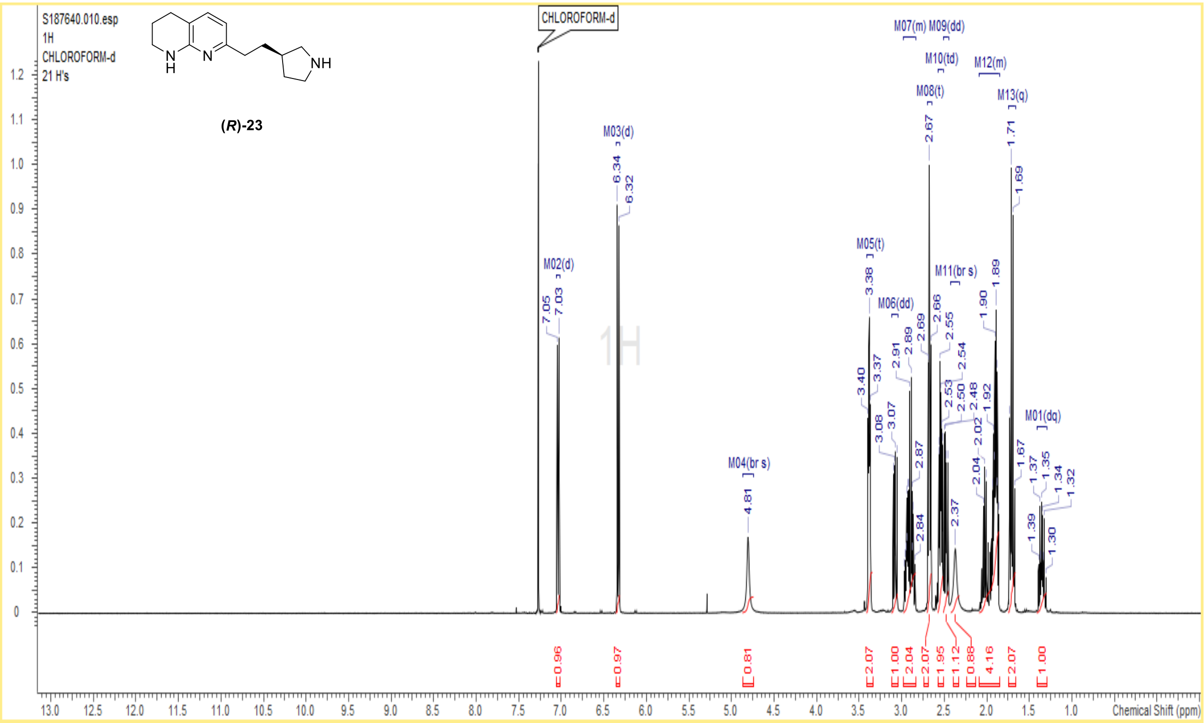
S187640.010.esp
1H
CHLOROFORM-d
21 H's
(R)-23
CHLOROFORM-d
M02(d)
7.05
7.03
M03(d)
6.34
6.32
M04(br s)
4.81
M05(t)
3.40
3.38
3.37
M06(dd)
3.08
3.07
2.91
2.89
2.87
2.84
M07(m) M09(dd)
M10(td)
M08(t)
2.69
2.67
2.66
2.55
2.54
2.53
2.50
2.48
2.37
M11(br s)
2.04
2.02
1.92
1.90
1.89
M12(m)
M13(q)
1.71
1.69
1.67
M01(dq)
1.39
1.37
1.35
1.34
1.32
1.30
0.96
0.97
0.81
2.07
1.00
2.04
2.07
1.95
1.12
0.88
4.16
2.07
1.00
1H
Chemical Shift (ppm)

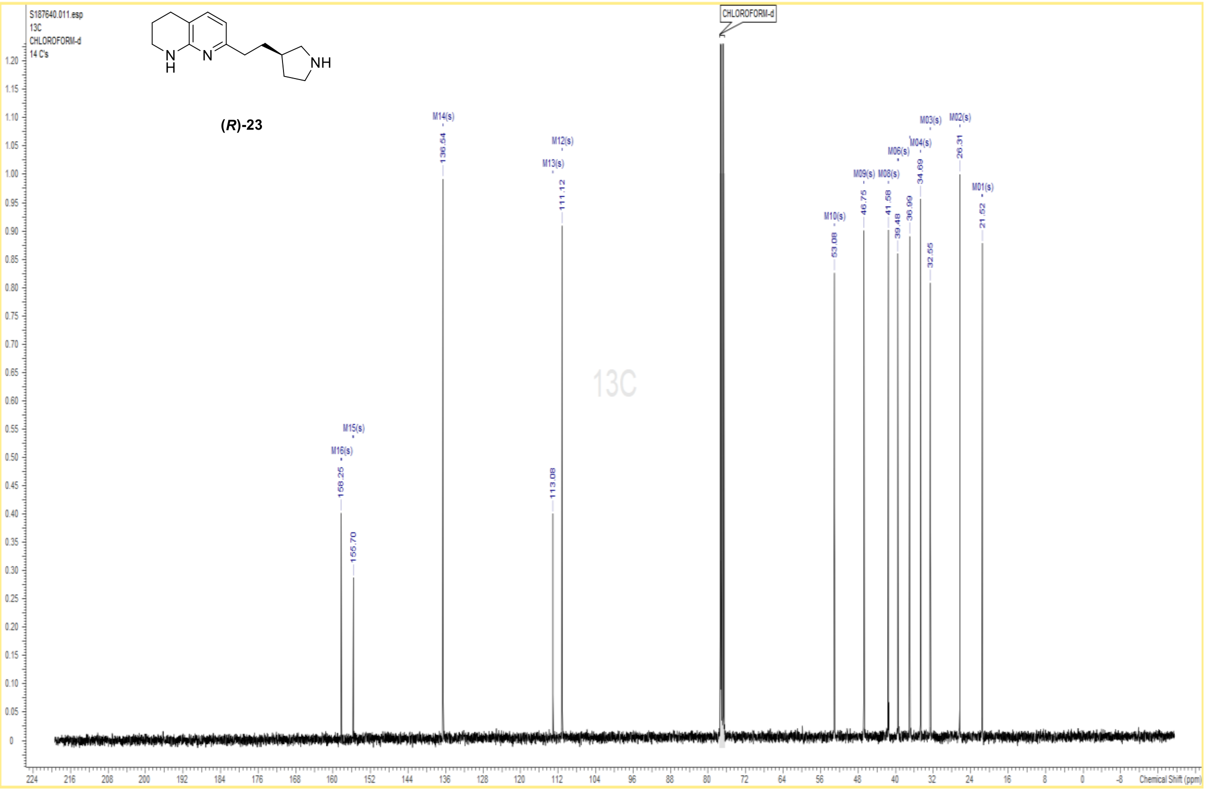
S187640.011.esp
13C
CHLOROFORM-d
14 C's
(R)-23
CHLOROFORM-d
M16(s)
158.25
M15(s)
155.70
M14(s)
136.54
M13(s)
113.08
M12(s)
111.12
M10(s)
53.08
M09(s)
46.75
M08(s)
41.58
39.48
M06(s)
36.99
M04(s)
34.69
M03(s)
32.55
M02(s)
26.31
M01(s)
21.52
13C
Chemical Shift (ppm)

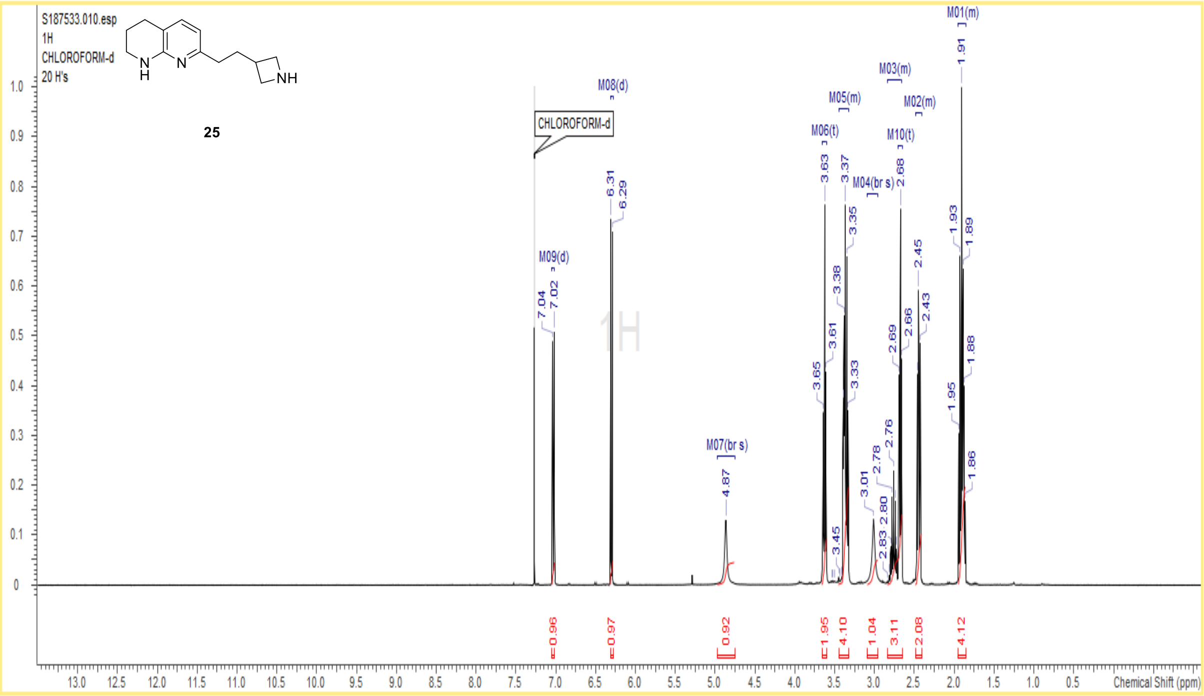
S187533.010.esp
1H
CHLOROFORM-d
20 H's
25
CHLOROFORM-d
Chemical Shift (ppm)

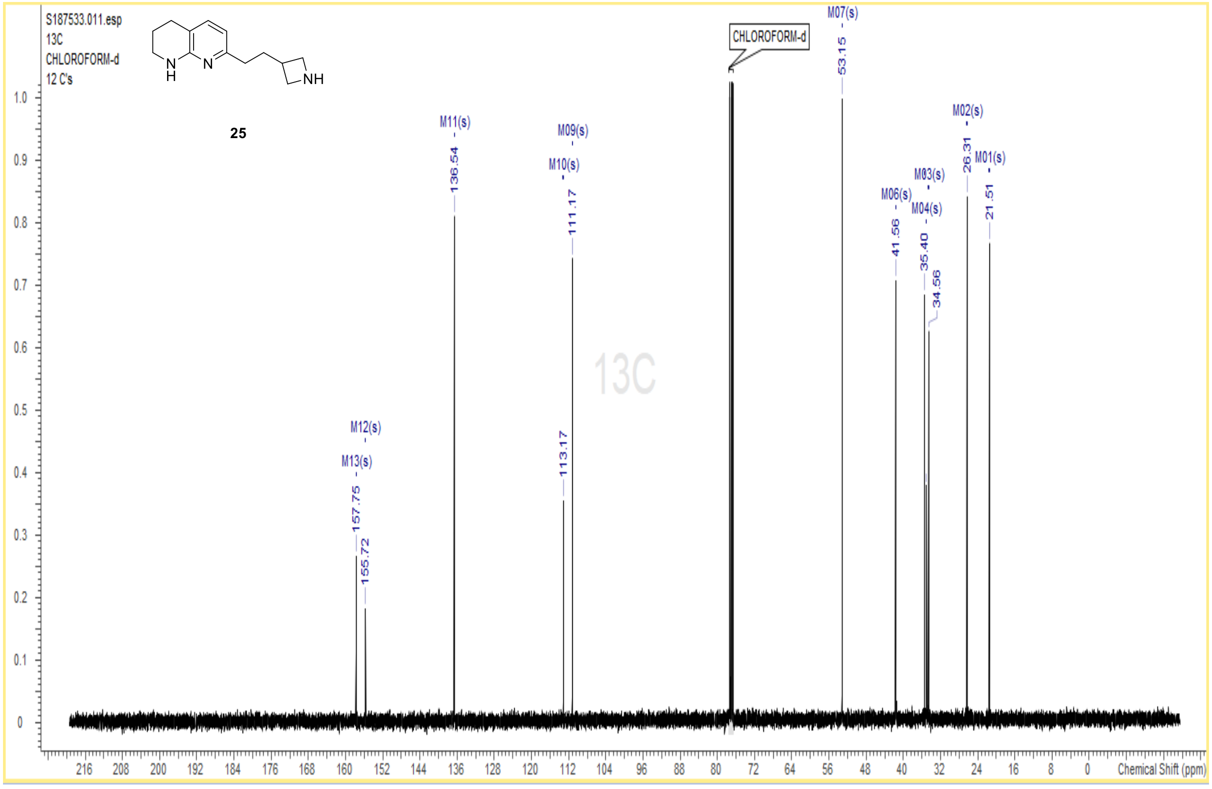
S187533.011.esp
13C
CHLOROFORM-d
12 C's
25
CHLOROFORM-d
Chemical Shift (ppm)

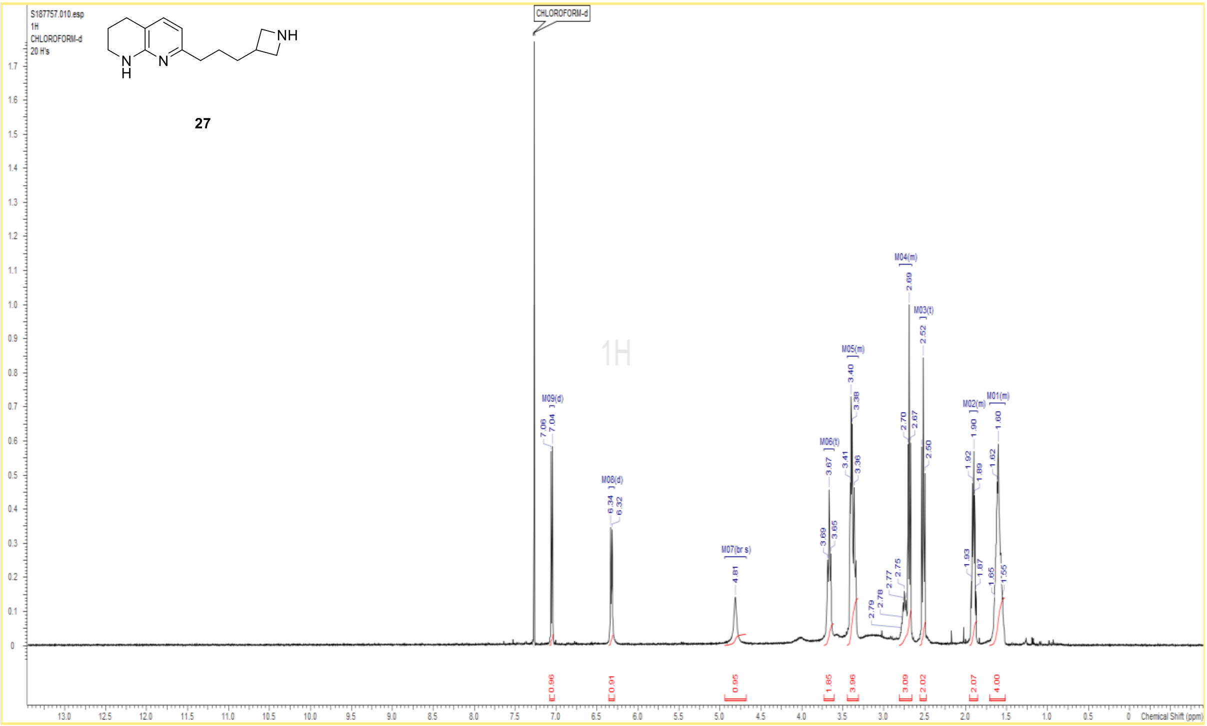
S167757.010.esp
1H
CHLOROFORM-d
20 H's
27
CHLOROFORM-d
Chemical Shift (ppm)

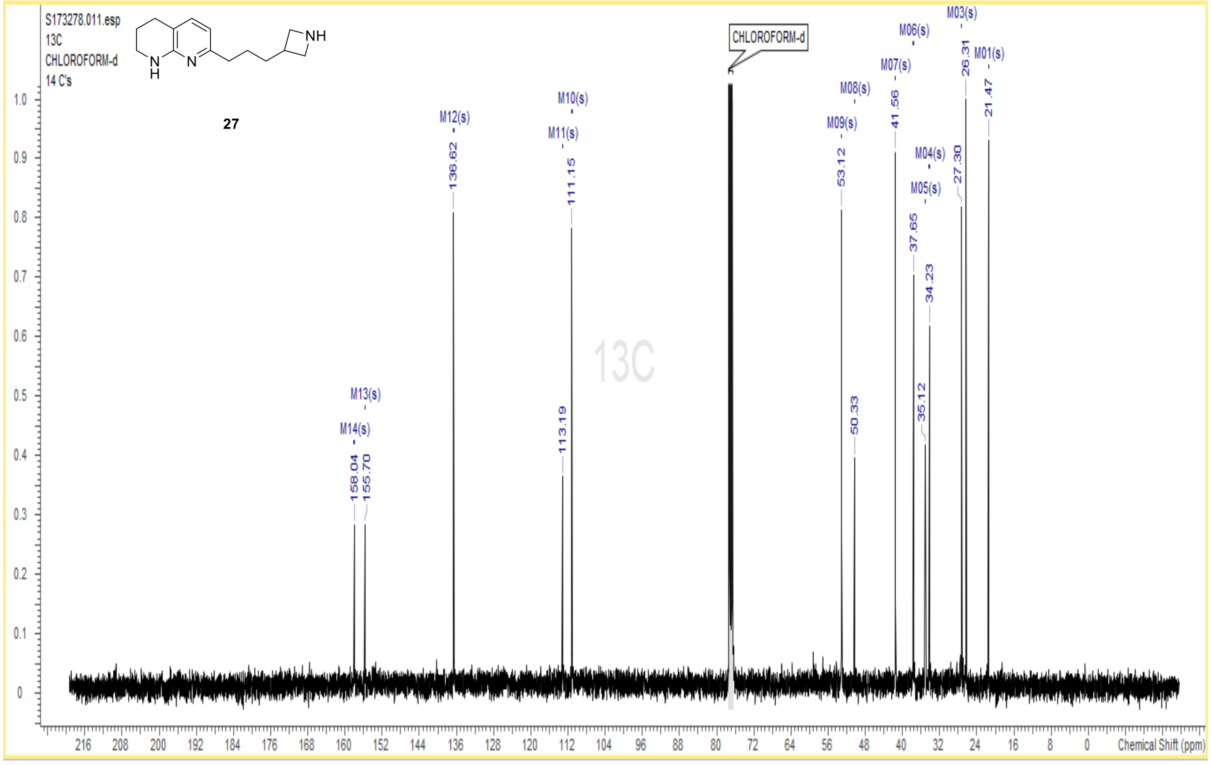
S173278.011.esp
13C
CHLOROFORM-d
14 C's
27
CHLOROFORM-d
Chemical Shift (ppm)

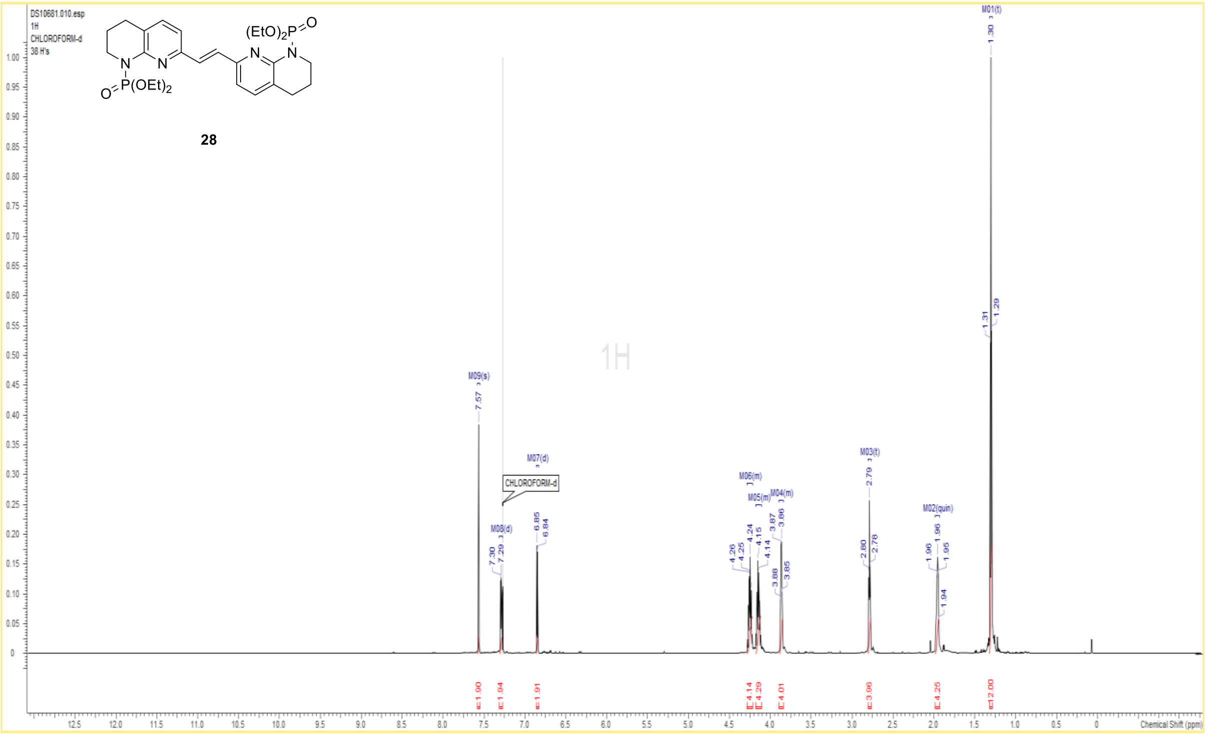
DS10681.010.esp
1H
CHLOROFORM-d
38 H's
(EtO)2P=O
O=P(OEt)2
28
1H
M09(s)
7.57
M08(d)
7.30
7.29
M07(d)
6.85
6.84
CHLOROFORM-d
M06(m)
M05(m)
M04(m)
4.26
4.25
4.24
4.15
4.14
3.87
3.86
3.85
M03(t)
2.80
2.79
2.78
M02(quin)
1.96
1.95
1.94
M01(t)
1.31
1.30
1.29
1.90
1.94
1.91
4.14
4.29
4.01
3.96
4.25
12.00
Chemical Shift (ppm)

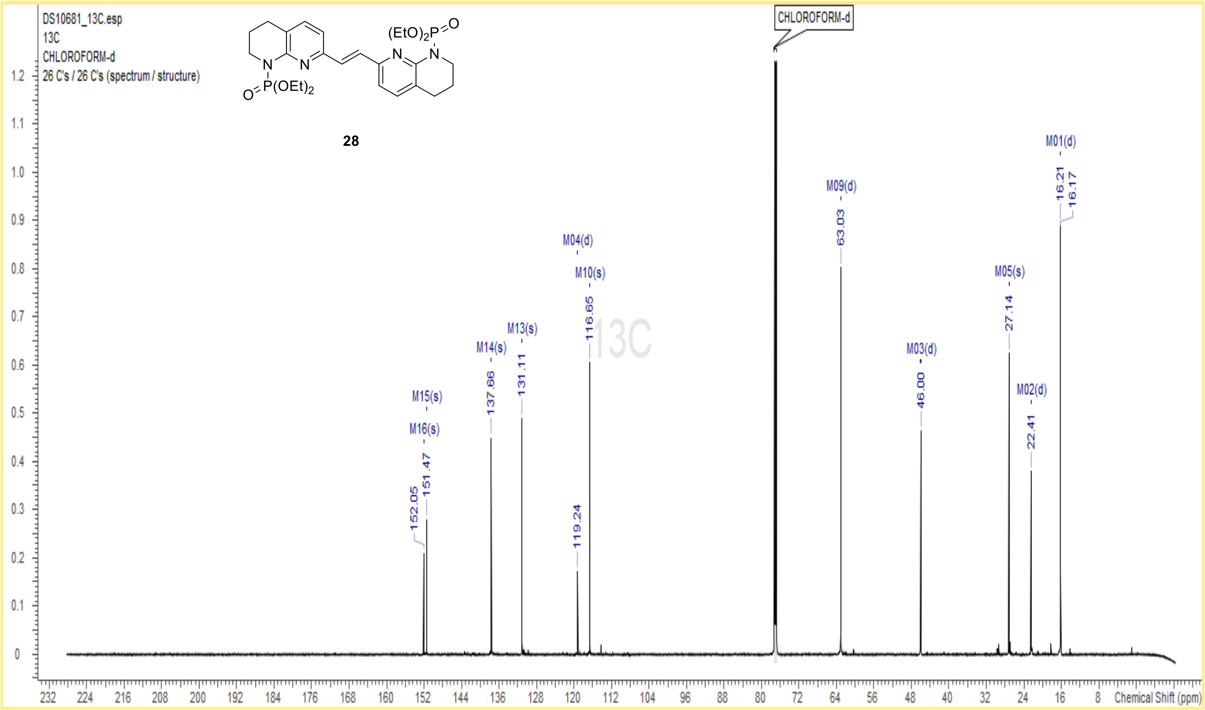
DS10681_13C.esp
13C
CHLOROFORM-d
26 C's / 26 C's (spectrum / structure)
(EtO)2P=O
O=P(OEt)2
28
13C
CHLOROFORM-d
M15(s)
M16(s)
152.05
151.47
M14(s)
137.66
M13(s)
131.11
M04(d)
119.24
M10(s)
116.65
M09(d)
63.03
M03(d)
46.00
M05(s)
27.14
M02(d)
22.41
M01(d)
16.21
16.17
Chemical Shift (ppm)

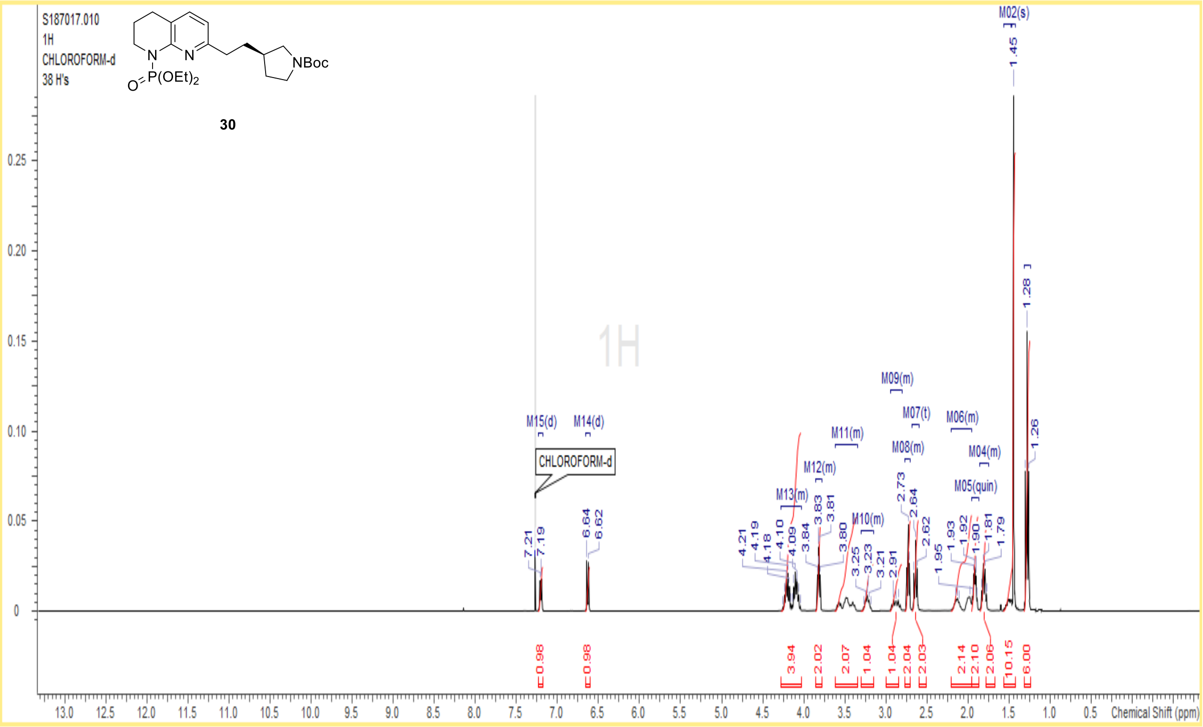

S187017.010
1H
CHLOROFORM-d
38 H's
30

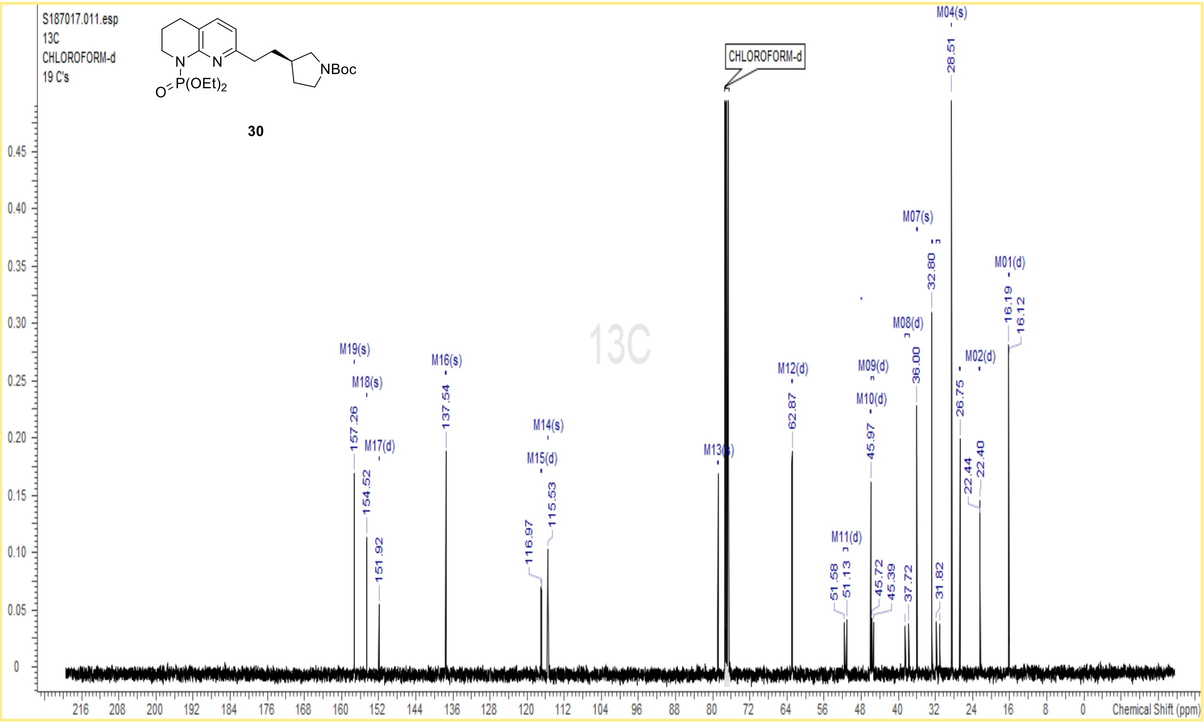

S187017.011.esp
13C
CHLOROFORM-d
19 C's
30

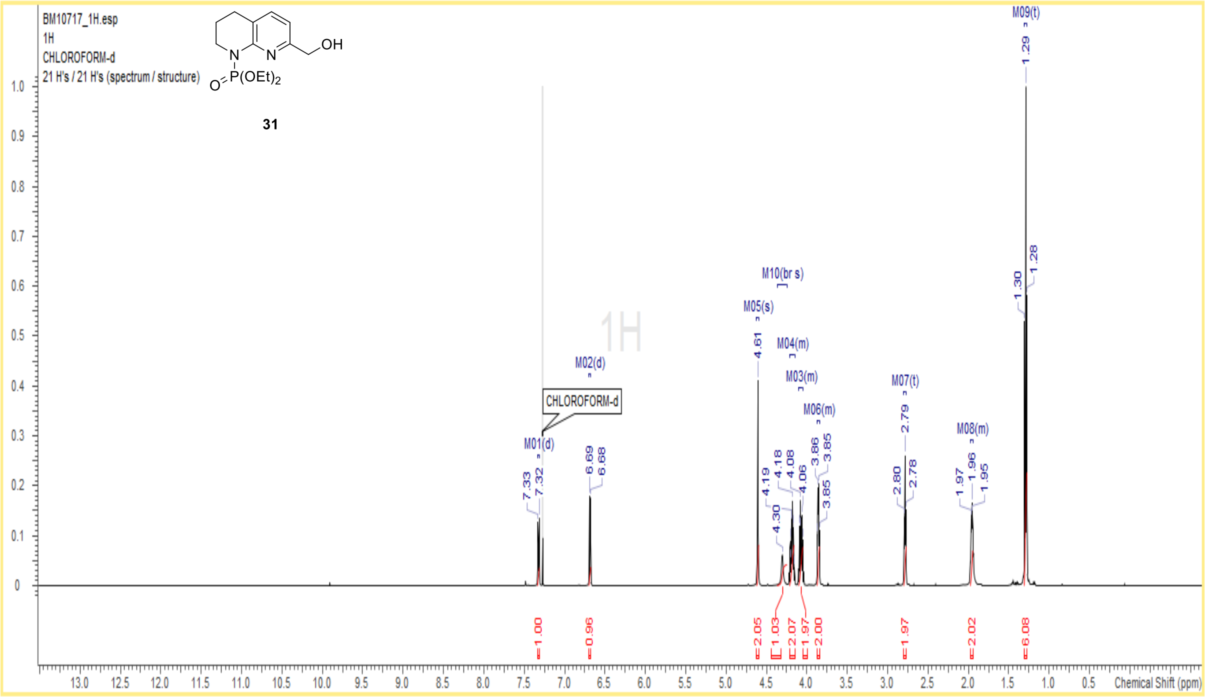
BM10717_1H.esp
1H
CHLOROFORM-d
21 H's / 21 H's (spectrum / structure)
31
CHLOROFORM-d
M01(d) 7.33 7.32
M02(d) 6.69 6.68
M05(s) 4.61
M10(br s)
M04(m)
M03(m)
M06(m)
M07(t) 2.80 2.79 2.78
M08(m) 1.97 1.96 1.95
M09(t) 1.30 1.29 1.28
Chemical Shift (ppm)

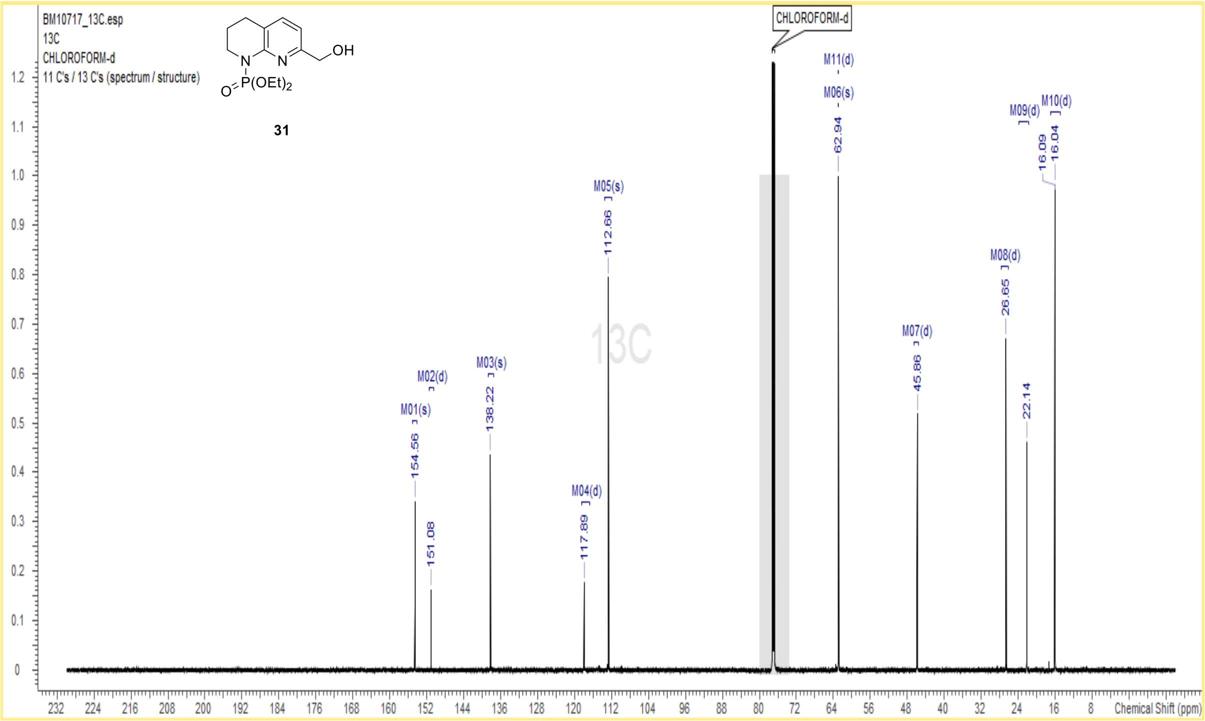
BM10717_13C.esp
13C
CHLOROFORM-d
11 C's / 13 C's (spectrum / structure)
31
CHLOROFORM-d
M01(s) 154.56
M02(d) 151.08
M03(s) 138.22
M04(d) 117.89
M05(s) 112.66
M11(d)
M06(s) 62.94
M07(d) 45.86
M08(d) 26.65
22.14
M09(d) 16.09
M10(d) 16.04
Chemical Shift (ppm)

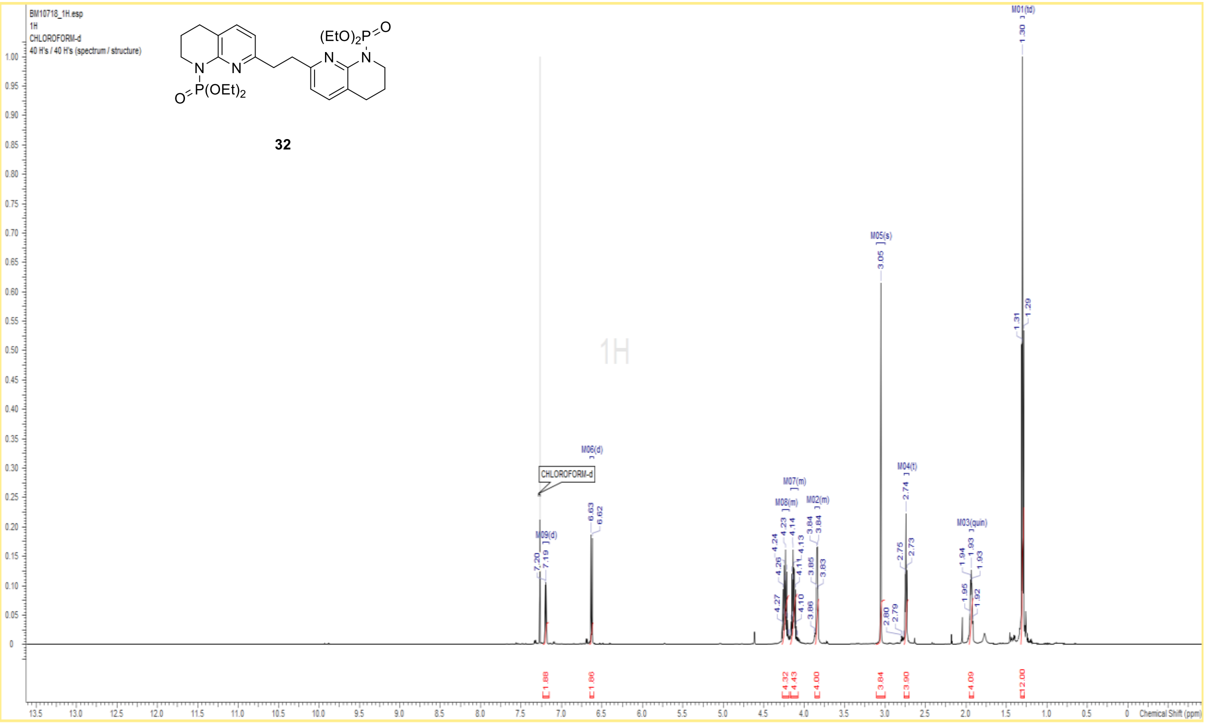
BM10718_1H.esp
1H
CHLOROFORM-d
40 H's / 40 H's (spectrum / structure)
(EtO)2P=O
O=P(OEt)2
CHLOROFORM-d
Chemical Shift (ppm)

32

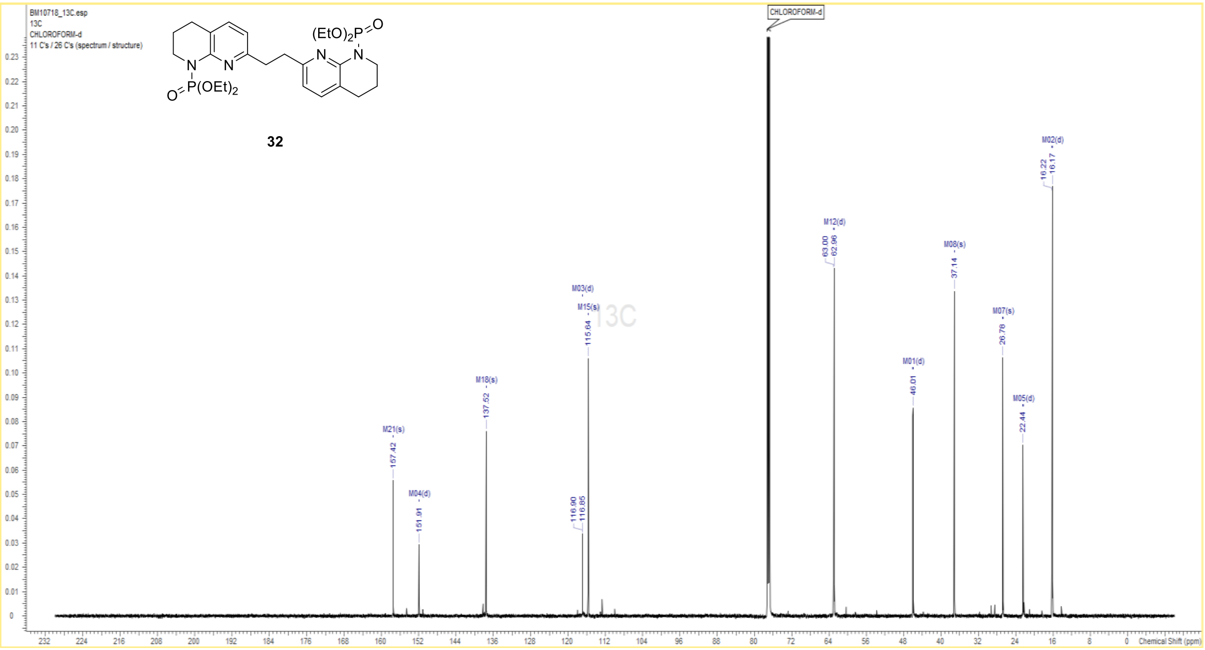
BM10718_13C.esp
13C
CHLOROFORM-d
11 C's / 26 C's (spectrum / structure)
(EtO)2P=O
O=P(OEt)2
CHLOROFORM-d
Chemical Shift (ppm)

32

Chiral HPLC analysis of compound 23 and (*R*)-23

<u>Sample obtained from HWE/reduction/deprotection route</u>

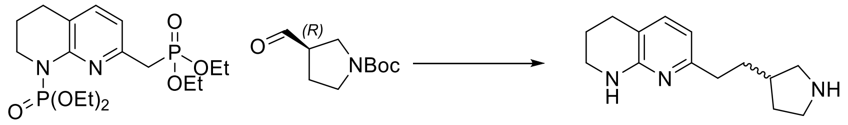

7 **(*R*)-22** **23**

In lieu of an available racemate, the tandem HWE/reduction/deprotection sequence was applied to the enantiomer (*S*)-**22** for use as a reference. The chromatograms are shown below: Top: mixture of samples; middle: sample obtained using (*S*)-**22** showing 24% ee; bottom: sample obtained using (*R*)-**22** showing 30% ee.

Method: Chiralpak ID (250 × 4.6 mm, 5 µm); flow rate: 1 mL/min; column temperature: 25 °C; mobile phase: heptane/ethanol/isopropylamine 70:30:0.1; run time: 30 min; detection: UV at 250 nm (band width 40 nm); injection: 5 µL of ethanol solution.

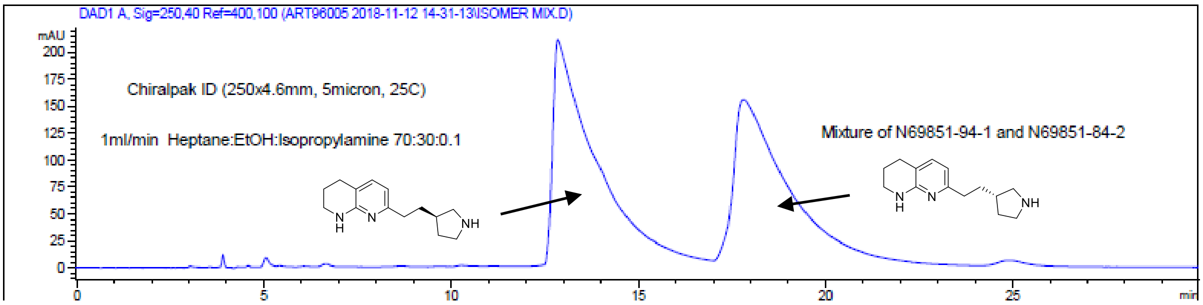

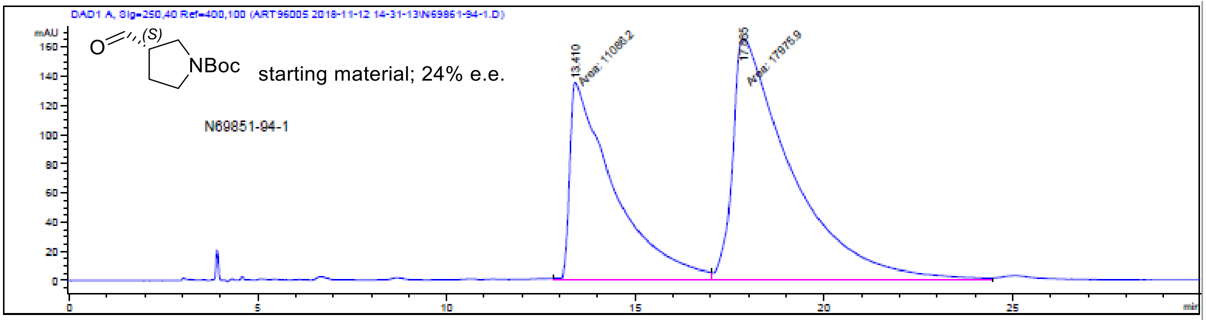

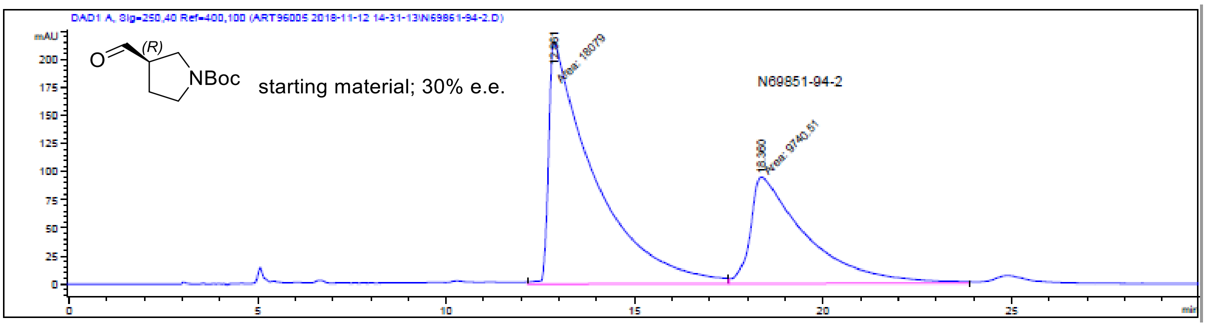

<u>Sample obtained from alkylation/deprotection route</u>

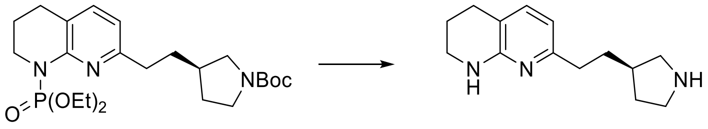

30 **(*R*)-23**

Method: Chiralpak ID (250 × 4.6 mm, 5 μm; flow rate: 1mL/min; column temperature = 25 °C; mobile phase: heptane/ethanol/isopropylamine 70:30:0.2; runtime: 20 min; detection: UV at 250 nm (bandwidth 40 nm); injection: 5 μL of ethanol solution.

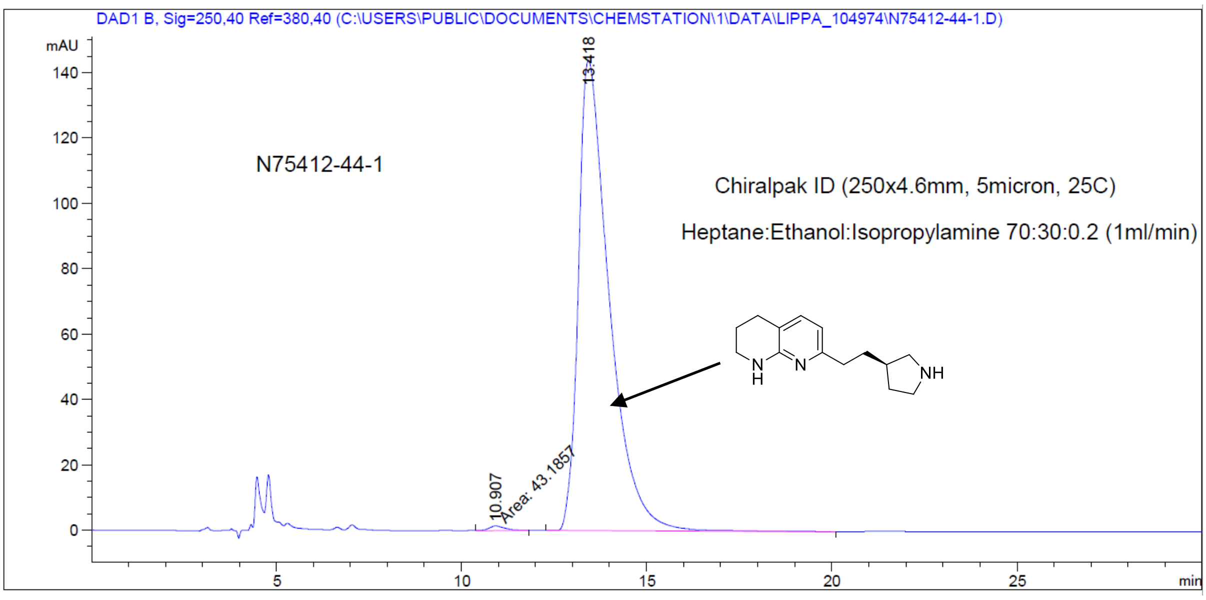
DAD1 B, Sig=250,40 Ref=380,40 (C:\USERS\PUBLIC\DOCUMENTS\CHEMSTATION\1\DATA\LIPPA_104974\N75412-44-1.D)
mAU
N75412-44-1
Chiralpak ID (250x4.6mm, 5micron, 25C)
Heptane:Ethanol:Isopropylamine 70:30:0.2 (1ml/min)
13.418
10.907
Area: 43.1857
NH
H
N
min
